# Natural Remedies for Gut Health: The Role of Plant Extracts in Combating Inflammatory Bowel Disease and Colon Cancer

**DOI:** 10.1002/fsn3.71403

**Published:** 2026-04-24

**Authors:** Oana‐Roxana Haralambie, Sanda Andrei

**Affiliations:** ^1^ Department of Preclinical Sciences, Faculty of Veterinary Medicine University of Agricultural Sciences and Veterinary Medicine Cluj‐Napoca Romania

**Keywords:** antioxidants, colon cancer, inflammatory bowel disease, oxidative stress, phytochemicals, plant extracts, traditional medicine

## Abstract

Digestive disorders such as inflammatory bowel disease (IBD) and colorectal cancer are among the most significant global health challenges, largely driven by chronic inflammation and oxidative stress. Over centuries, traditional medical systems have relied on medicinal plants to alleviate gastrointestinal conditions, and current pharmacological research is beginning to validate these ancestral practices. Many plants contain a rich diversity of bioactive compounds, including polyphenols, flavonoids, terpenes, and alkaloids, that exert antioxidant, anti‐inflammatory, and anticancer effects, making them promising candidates for complementary therapy. This review compiles and critically analyzes data from the past decade on plant extracts and isolated natural compounds tested in vitro, in vivo, and ex vivo for their protective effects on intestinal health. Evidence consistently indicates that plant‐derived molecules can suppress inflammatory mediators, modulate oxidative stress responses, restore epithelial barrier integrity, and induce apoptosis in neoplastic cells. Among these, polyphenols and flavonoids emerge as key contributors, demonstrating efficacy across multiple biological models. The findings support the growing integration of traditional herbal knowledge with modern biomedical science. Plant‐based compounds represent not only valuable sources of therapeutic agents but also a foundation for developing functional foods and nutraceuticals aimed at reducing inflammation and preventing colon‐related pathologies.

## Graphical abstract

Created in BioRender. Haralambie, O. (2025) https://BioRender.com/dh5ly89.

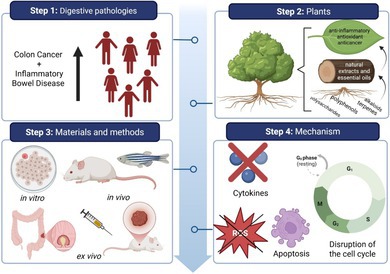

**Abbreviations**
5FU: 5‐Fluorouracil (chemotherapy drug)
5‐HT: 5‐Hydroxytryptamine (Serotonin)
A549: human lung carcinoma cell line (non‐small cell lung cancer)
ABTS: assay to measure free radical scavenging activity
ACE: aronia crude extract
ADA: adenosine deaminase
Akt: protein kinase B
ALP: alkaline phosphatase
ALT: alanine aminotransferase
APC: adenomatous polyposis coli
APE: aronia purified extract
ASC: apoptosis‐associated speck‐like protein containing a CARD
AST: aspartate aminotransferase
ATF4: activating transcription factor 4
BALB/c: Bagg Albino Laboratory mouse strain used in research
Bax: Bcl‐2‐associated X protein (proapoptotic protein)
Bcl‐2: B‐cell lymphoma 2 (anti‐apoptotic protein)
BJ: human fibroblast cell line (non‐cancerous)
C57BL/6: common inbred mouse strain
Caco‐2: human colorectal adenocarcinoma cell line
Caspase‐3: enzyme playing a key role in apoptosis
CAT: catalase
CCD‐18: human colonic myofibroblast cell line
CD: Crohn's disease
CD‐1 nu/nu: immunocompromised mouse strain
CD4+ T cells: helper T cells with CD4 receptor
CDK2, CDK4: cyclin‐dependent kinases 2 and 4
CIE: chronic inflammatory enteropathy
C‐myc: cellular myelocytomatosis oncogene
Colo320: human colon carcinoma cells
COX‐2: cyclooxygenase‐2
CRC: colorectal cancer
CSCs: cancer stem cells
CT26: murine colon carcinoma cell line
Cyclin D1: cell cycle protein regulating G1/S transition
DAI: disease activity index
DLD‐1: human colorectal adenocarcinoma cell line
DMSO: dimethyl sulfoxide
DNA: deoxyribonucleic acid
DPPH: 2,2‐diphenyl‐1‐picrylhydrazyl
dROM: derivatives of reactive oxygen metabolites
DSS: dextran sulfate sodium
E2F1: transcription factor 1
EGF: epidermal growth factor
EGFR: epidermal growth factor receptor
eNOS: endothelial nitric oxide synthase
ERK1/2: extracellular signal‐regulated kinases 1 and 2
ex vivo Latin: “out of the living” (experiments on tissues outside the organism but in an environment mimicking the natural conditions)
FHC: human normal colon epithelial cell line
FNDF‐ABJ: fermented non‐digestible fraction of Andean berry juice
FRAP: Ferric reducing antioxidant power
FV: *Fagiola di Venanzio*
G0/G1 phase: resting/first gap phase of cell cycle
G1 arrest: cell cycle arrest in the G1 phase
G2/M phase: Gap 2/mitosis phase of the cell cycle
GAE: Gallic acid equivalent (antioxidant assay unit)
GGT or γ‐GT: Gamma‐glutamyl transferase
GPX2: glutathione peroxidase 2
GSDMD: Gasdermin D
GSH: glutathione
GSH‐PX: glutathione peroxidase
GSK3β: glycogen synthase kinase‐3 beta
H_2_O_2_
: hydrogen peroxide
HCEC‐1CT: human colon epithelial cell line
HCT‐116: human colorectal carcinoma cell line
HCT‐15: human colon adenocarcinoma cell line
HCT‐8: human colon carcinoma cell line
HIF‐1α: hypoxia‐inducible factor 1‐alpha
HO‐1: heme oxygenase‐1
HSkMC: human skeletal muscle cells, fibroblast‐like (control cells)
HT‐29: human colon adenocarcinoma cell line
HTB‐39: human colon adenocarcinoma cells
IBD: inflammatory bowel disease
IC_50_
: half maximal inhibitory concentration
IFN‐γ: interferon gamma
IGF‐1R: insulin‐like growth factor‐1 receptor
IL‐10: interleukin‐10
IL‐12: interleukin‐12
IL‐13: interleukin‐13
IL‐17: interleukin‐17
IL‐1β: interleukin‐1 beta
IL‐2: interleukin‐2
IL‐4: interleukin‐4
IL‐5: interleukin‐5
IL‐6: interleukin‐6
IL‐8: interleukin‐8
iNOS: inducible nitric oxide synthase
in vitro Latin: “in glass” (experiments performed outside a living organism)
in vivo Latin: “within the living” (experiments performed in living organisms)
JNK: c‐Jun N‐terminal kinase
K‐ras: Kirsten rat sarcoma viral oncogene homolog
Kunming mice: mouse strain used in research
LC3: autophagy‐related protein
LC3II: autophagy marker
LDH: lactate dehydrogenase
LNCaP: human prostate cancer cell line
LOOH: lipid hydroperoxides
LOX‐5 (5‐LOX): 5‐lipoxygenase enzyme
LPS: lipopolysaccharide
MAPK: mitogen‐activated protein kinase
MC‐38: mouse colon adenocarcinoma cells
MCF7: human breast cancer cell line
MDA: malondialdehyde
mg/kg: milligrams per kilogram (body weight)
miR‐34a: microRNA involved in tumor suppression
miRNA: microRNA
MMP, MMP‐2, MMP‐9: matrix metalloproteinases
MPO: myeloperoxidase
mRNA: messenger ribonucleic acid
mTOR: mechanistic target of rapamycin
NAG‐1: NSAID‐activated gene 1
NCM460: normal human colon mucosal epithelial cell line
NCR nu/nu nude mice: immunodeficient mouse strain used for xenografts
NFE2L2 (Nrf2): nuclear factor erythroid 2–related factor 2
NF‐κB: nuclear factor kappa‐light‐chain‐enhancer of activated B cells
NLRP3: NOD‐, LRR‐, and pyrin domain‐containing protein 3
NO: nitric oxide
NOD/SCID: non‐obese diabetic/severe combined immunodeficient mice
Notch1, Notch2: oncogenes in cell differentiation/proliferation
NOX1: NADPH oxidase 1
Nrf2: nuclear factor erythroid 2–related factor 2
nu/nu (nude): athymic nude mice
ORAC: oxygen radical absorbance capacity
OS: oxidative stress
OSI: oxidative stress index
p38 MAPK: p38 mitogen‐activated protein kinase (signaling pathway)
p53: tumor protein p53
PARP: poly(ADP‐ribose) polymerase
PBS: phosphate‐buffered saline
PCNA: proliferating cell nuclear antigen
P‐c‐Raf: phosphorylated c‐Raf
PDK1: 3‐phosphoinositide‐dependent protein kinase‐1
PDX: patient‐derived xenograft
PGE2: prostaglandin E2
PI3K: phosphoinositide 3‐kinase
PIAS3: protein inhibitor of activated STAT 3
PIK3CA: phosphatidylinositol‐4,5‐Bisphosphate 3‐Kinase Catalytic Subunit Alpha
p.o.: Per os (oral administration)
PSA: prostate‐specific antigen
PTEN: phosphatase and tensin homolog
PUMA: p53 upregulated modulator of apoptosis
RAW264.7: murine macrophage cell line
Rb: Retinoblastoma Protein
RKO: human colorectal carcinoma cell line
RNS: reactive nitrogen species
RORγt: RAR‐related orphan receptor gamma
ROS: reactive oxygen species
SASA: Sasa quelpaertensis
SCFA: short‐chain fatty acids
SCID: severe combined immunodeficient (mouse model)
SH: sulfhydryl group
SN‐38: active metabolite of irinotecan
SOD: superoxide dismutase
SPF: specific pathogen‐free
STAT3: signal transducer and activator of transcription 3
SW‐480: human colon adenocarcinoma cell line
T‐84: human colon adenocarcinoma cell line
TBARS: thiobarbituric acid reactive substances
TCIPA: tumor cell‐induced platelet aggregation
TGF‐β: transforming growth factor‐beta
Th1: T helper type 1
Th17/Treg axis: balance between Th17 and Treg cells
Th17 cells: pro‐inflammatory T helper cells
TLR‐4: toll‐like receptor 4
TNBS: 2,4,6‐Trinitrobenzenesulfonic acid
TNF‐α: tumor necrosis factor‐alpha
TRAIL‐R2: TNF‐related apoptosis‐inducing ligand receptor 2
U‐87: human glioblastoma cell line
UC: ulcerative colitis
Wnt/β‐Catenin: cell proliferation/cancer signaling pathway
ZO‐1: Zonula occludens‐1 (Tight junction protein)
β‐sitosterol: plant phytosterol
μg/mL: micrograms per milliliter

## 1 Introduction

The intestinal epithelium is characterized by an exceptionally high rate of cellular renewal; however, this regenerative capacity is markedly impaired under chronic inflammatory conditions (Parker et al. 2019). Inflammatory bowel diseases (IBDs), encompassing Crohn's disease (CD) and ulcerative colitis (UC), represent chronic, relapsing inflammatory disorders of the gastrointestinal tract (Tinnirello et al. 2024). UC, in particular, is histopathologically defined by widespread mucosal ulceration, intense neutrophil infiltration, and epithelial necrosis (Bouayad Debbagh et al. 2023).

Both major forms of IBD are characterized by persistent cycles of inflammation and remission (Kölbel et al. 2024), predominantly affecting the colonic mucosa and submucosa. These processes are driven by the hyperactivation of immune cells and excessive secretion of pro‐inflammatory cytokines (Gu et al. 2024; Machado et al. 2021; Pervin et al. 2016; Zielińska et al. 2015). Although the precise etiology of IBD remains incompletely understood (Tinnirello et al. 2024), the disease appears to arise from complex interactions among genetic predisposition, intestinal dysbiosis, immune dysregulation, and impairment of the mucosal barrier (Bibi et al. 2018).

Persistent inflammation and immune imbalance also contribute to the heightened risk of colorectal cancer (CRC) in IBD patients (Maurer et al. 2020), making CRC one of the most serious long‐term complications and a major cause of morbidity and mortality in this population (McFadden et al. 2015). Chronic mucosal inflammation (McIntyre et al. 2015) and alterations in the intestinal microbiota (Tomkovich et al. 2017) are believed to play key roles in this malignant transformation. For over a decade, accumulating evidence has underscored the combined contribution of environmental exposures, persistent inflammation, and microbial dysbiosis to tumorigenesis by promoting adenoma formation and progression toward carcinoma (Caron et al. 2024; McFadden et al. 2015).

Furthermore, oxidative stress and dietary mutagens are recognized as important cofactors in the pathogenesis of colorectal neoplasia. Diets rich in saturated fats have been strongly associated with increased oxidative DNA damage and a higher risk of developing colorectal malignancies (Pedro et al. 2016). Collectively, these findings highlight the multifactorial nature of IBD and its close pathophysiological and etiological connection to CRC development.

### 1.1 Incidence and Epidemiology

UC and CD represent major gastroenterological disorders with increasing incidence and prevalence, particularly in industrialized nations (Peng et al. 2019). Recent epidemiological data indicate substantial regional variation in IBD prevalence, ranging from 187 to 832 per 100,000 individuals in Europe, 1.83–70.1 per 100,000 in South America, and 214.9–478.4 per 100,000 in North America (Caron et al. 2024). By 2030, the number of patients affected by IBD, including both CD and UC, is projected to rise significantly, with the steepest increases occurring among pediatric and elderly populations (Caron et al. 2024).

This upward trend is partially attributed to improved therapeutic strategies that have enhanced patient survival; however, a concurrent rise in IBD‐associated CRC has also been observed (Kim et al. 2015). In 2023, CRC ranked as the third most commonly diagnosed malignancy worldwide, accounting for ~10% of all cancer cases, and remained the second leading cause of cancer‐related mortality (“World Health Organization” 2025). The highest incidence rates are reported in Europe, Australia, and New Zealand, while Eastern Europe exhibits the greatest mortality burden (“World Health Organization” 2025). Similarly, countries in Latin America and the Caribbean, such as Brazil, Chile, and Costa Rica, have shown increasing incidence and mortality trends in recent years (Nascimento et al. 2023).

In Romania, colon cancer represented the most frequently diagnosed malignancy in 2022, ranking first overall, second among women, and third among men (“GLOBOCAN” 2025). Projections suggest that by 2040, the global incidence of CRC will reach ~3.2 million new cases annually, with 1.6 million associated deaths (“World Health Organization” 2025). Age remains one of the primary risk factors, as the majority of cases occur in individuals over 50 years old (“World Health Organization” 2025).

In the veterinary field, chronic inflammatory enteropathies (CIE) in dogs have been reported with a prevalence of ~2% (Sahoo et al. 2023). Although their etiology remains multifactorial and not yet fully understood, these conditions share several pathogenic similarities with human IBD (Sahoo et al. 2023). Moreover, CRC has been documented in various animal species, including sheep and dogs, with incidence rates differing according to species and environmental exposure (Acquaviva et al. 2016).

### 1.2 Oxidative Stress in Intestinal Pathologies

The generation of reactive oxygen species (ROS) is a fundamental component of normal cellular metabolism. Under physiological conditions, ROS contribute to essential biological processes such as pathogen elimination, wound healing, and tissue regeneration (Sahoo et al. 2023). They also serve as critical signaling molecules that regulate cell proliferation, migration, and differentiation (Andrei et al. 2014; Jelic et al. 2021).

Although the precise etiology of IBDs remains unclear, oxidative stress is widely recognized as a major pathogenic factor contributing to intestinal inflammation (Abdullah Al‐Omran et al. 2023; Chen et al. 2024). This imbalance results from excessive production of ROS and reactive nitrogen species (RNS), which overwhelms endogenous antioxidant defenses (Adjouzem et al. 2020; Rytsyk et al. 2020). In colitis, this redox dysregulation leads to extensive lipid peroxidation, DNA fragmentation, and protein oxidation in intestinal tissues (Adjouzem et al. 2020).

Experimental evidence further supports the role of oxidative stress in gastrointestinal pathology. For instance, Candellone et al. (2022) compared oxidative stress profiles between dogs with acute, uncomplicated diarrhea and healthy controls (Candellone et al. 2022). The affected animals exhibited elevated levels of reactive oxygen metabolites (dROMs) and a higher oxidative stress index (OSI), along with reduced total antioxidant capacity. These findings highlight the role of systemic redox imbalance in contributing to inflammatory processes within the gut.

Oxidative damage not only exacerbates intestinal inflammation but also represents a key early event in mutagenesis and carcinogenesis (Carini 2017). Persistent ROS and RNS exposure can induce DNA mutations, disrupt signaling pathways, and activate oncogenic transcription factors, ultimately predisposing cells to neoplastic transformation.

### 1.3 Current Treatment Options in Inflammatory Bowel Disease and Colon Cancer

The therapeutic management of IBD has evolved significantly over the past decade. Earlier approaches, such as antibiotic, probiotic, and antitumor necrosis factor‐alpha (TNF‐α) therapies, were commonly employed to control intestinal inflammation (Zielińska et al. 2015). Currently, standard treatment strategies rely primarily on anti‐inflammatory agents, including corticosteroids, 5‐aminosalicylates, and immunosuppressants, which effectively reduce inflammation and relieve symptoms (Peng et al. 2019; Tinnirello et al. 2024).

Despite their clinical benefits, these conventional therapies are often associated with substantial long‐term adverse effects (Peng et al. 2019; Saxena et al. 2014). Prolonged corticosteroid use may lead to hepatic and renal toxicity (Peng et al. 2019), hyperglycemia, muscle atrophy, and ocular complications such as glaucoma (Adjouzem et al. 2020). Moreover, the emergence of drug resistance and hypersensitivity reactions further complicates chronic disease management (Machado et al. 2021).

For CRC, conventional therapeutic protocols typically involve surgical resection of the primary tumor, and when indicated, adjuvant radiotherapy or chemotherapy, particularly in advanced or metastatic disease (Nascimento et al. 2023; “World Health Organization” 2025). Although these interventions remain the cornerstone of CRC management, they are often accompanied by severe systemic side effects, reduced patient quality of life, and variable treatment efficacy (Bar‐Shalom et al. 2019; Nascimento et al. 2023).

Overall, while conventional pharmacological and oncological approaches have improved patient survival and symptom control, their limitations underscore the need for complementary or alternative strategies, particularly those based on bioactive natural compounds with anti‐inflammatory and antioxidant potential.

### 1.4 Traditional Use of Medicinal Plants in the Treatment of Inflammatory Bowel Disease and Colon Cancer

Before the emergence of modern pharmacological therapies, medicinal plants played a central role in managing gastrointestinal disorders, including IBD and colon cancer. Traditional systems of medicine, particularly Traditional Chinese Medicine (TCM), frequently relied on plant‐derived bioactive compounds to alleviate inflammation, oxidative stress, and intestinal dysbiosis (Yutong Jin et al. 2025). Various plant parts, such as leaves, roots, seeds, and fruits, were used depending on the targeted ailment and desired therapeutic effect (Aiello et al. 2019).

According to a comprehensive review by Aiello et al. (2019), several plants commonly used in traditional medicine, including grape, soybean, green tea, garlic, olive, and pomegranate, exhibit strong chemopreventive and anti‐inflammatory potential (Aiello et al. 2019). Their activity is largely attributed to high concentrations of polyphenols, polysaccharides, and triterpenoids, which act through mechanisms involving the reduction of oxidative stress, modulation of the gut microbiota, and regulation of intestinal immune pathways (Yutong Jin et al. 2025). Although most studies confirm their general safety, some have reported variability in efficacy or potential adverse reactions, underscoring the need for standardized dosing and rigorous toxicological evaluation (Aiello et al. 2019; Rohani et al. 2022).

A notable example of the therapeutic potential of traditional plant‐based remedies is *Phaseolus vulgaris* L., known as “Fagiola di Venanzio” (FV), a rare Italian bean variety (Bernardi et al. 2024). In vitro intestinal cell models demonstrated that extracts derived from soaking water, cooking water, and bioaccessible fractions obtained through simulated digestion effectively attenuated interleukin‐1β‐induced inflammation. This effect was associated with reduced cyclooxygenase‐2 (COX‐2) expression, lower prostaglandin E2 levels, and decreased production of reactive oxygen species (ROS) via downregulation of the NOX1 enzyme (Bernardi et al. 2024).

Similarly, *Rubus adenotrichos* (blackberry), traditionally consumed in Central America, is a rich source of polyphenols and anthocyanins that exhibit antineoplastic potential. Extracts from ripe fruits, pulp, and filtered juices demonstrated cytotoxic effects against the SW‐620 human colorectal adenocarcinoma cell line, with an IC_50_ of 112 μg/mL (Madrigal‐Gamboa et al. 2021). Another species, *Vaccinium meridionale* Swartz (Andean berry), also rich in anthocyanins and phenolic acids, showed proapoptotic activity in HT‐29 colon adenocarcinoma cells. Its fermented non‐digestible juice fraction (FNDF‐ABJ), obtained from freeze‐dried powder, induced apoptosis through oxidative stress mechanisms, evidenced by DNA fragmentation and reduced superoxide dismutase (SOD) activity (Agudelo et al. 2020).

This review provides a comprehensive summary of recent natural products exhibiting significant activity against IBD and colon cancer. The therapeutic use of traditional medicinal plants provides a valuable foundation for identifying novel bioactive compounds with relevance to IBD and colon cancer. Continued research into these phytonutrients may facilitate the development of safe, effective, and sustainable alternatives or complementary therapies for the prevention and management of chronic intestinal diseases.

## 2 Sources and Methodology

All data were gathered through an extensive search of electronic databases, including Web of Science Core Collection, Wiley Journals, Science Direct, Policy Commons, PubMed, SCOPUS, and Google Scholar, with the search restricted to English‐language publications. Keywords used in the search included “inflammatory bowel disease in vitro,” “inflammatory bowel disease in vivo,” “colon cancer in vitro,” “colon cancer in vivo,” traditional medicine, cancer phytotherapy, plant extracts in IBD/colon cancer, antioxidants, antitumoral natural substances, botanical extracts with anticancer properties, natural products against cancer and oxidative stress in IBD/colon cancer. Most of the original articles were accessed, and relevant data were extracted. The first step of the review involved analyzing the titles and abstracts of the retrieved articles, with the criterion that eligible articles were published between 2015 and 2025. Many studies on our topic of interest were found within this period. The second step examined the full text of eligible articles, which had to be available for reading or purchase. Detailed information on the plants studied, their biochemical characterization, and any in vitro or in vivo tests on their antiproliferative effects on colon cancer cells or IBD was reported. All final articles had to include references and be cited at least once, except those published in the past 6 months. Citation counts were determined using the Web of Science Core Collection search tool, with Google Scholar used when articles were unavailable.

Table 1 presents data from over 40 studies that evaluated 55 different medicinal plants for their protective effects against IBD using in vitro and in vivo experimental models. The plants represent a wide botanical diversity and contain major classes of phytochemicals, such as polyphenols, flavonoids, terpenes, alkaloids, and iridoid glycosides. Extracts were prepared with ethanol, methanol, water, or hydroalcoholic solvents and tested at concentrations ranging from micrograms in cell‐based assays to grams per kilogram in animal models.

**TABLE 1:** Bioactive compounds tested against inflammatory bowel disease (IBD).

Nr.	Plant name	Common name	Part used	Most important bioactive components	Administration/type of extract	Dose concentration	Mechanism	References
Colitis								
In vitro								
Multiple classes of compounds								
1.	*Lens culinaris var. Eston green*	Lentil	Lentils	Alkaloids, Aminoacids, Peptides, Phenolic Compounds	Ethanolic Extract, Ethyl Acetate Extract	0.25–100 μg/mL	↓ iNOS expression, ↓TLR‐4 expression, ↓ pro‐inflammatory cytokine IL‐1 production, ↑ anti‐inflammatory cytokine IL‐10 production, ↑ cell viability	Panaro et al. (2024)
In vivo								
Polyphenols								
2.	*Pyrus elaeagnifolia*	Oleaster‐Leaved Pear	Fruits	Phenolic Compounds, Tannin, and Flavonoid	Methanolic Extract	100 mg/kg	↓ MDA, nitrite, IL‐6, and TNF‐α levels in the colon tissue and anti‐inflammatory properties	Ilhan et al. (2019)
3.	*Aronia mitschurinii*	Aronia Berry, Black Chokeberry	Fruits	Polyphenols, Proanthocyanidins	Aqueous Extract	4.5% Aronia supplemented	↓ T cell TNF‐α secretion, ↑ colonic IL‐10	Martin et al. (2018)
Flavonoids								
4.	*Lycium ruthenicum*	Black Goji	Fruits	Anthocyanins	Ethanolic Extract	200 mg/kg/day	↓ pro‐inflammatory cytokines and related mRNA (TNF‐α, IL‐6, IL‐1β and IFNγ), and ↑proteins, such as ZO‐1, occludin and claudin‐1, ↓ NF κB in the front end	Peng et al. (2019)
5.	*Achyrocline satureioides*	Macela	Inflorescences	Flavonoids Luteolin and Quercetin	Hydroalcoholic Extract	1, 10, 100 mg/kg	↓ Inflammatory cytokines and oxidative mediators, ↑ IL‐4 and IL‐10	Da Silva et al. (2016)
6.	*Prunus mahaleb*	Mahaleb Cherry	Fruits	Flavonoids (Anthocyanins)	Extract	25 μL p.o	↑ Mitochondrial oxidative metabolism via the activation of the Nrf2 pathway, ↑ antioxidant defenses	Ferramosca et al. (2019)
Alkaloids								
7.	*Taraxacum campylodes* , *Coix lachryma‐jobi*, *Smilax glabra Roxb*, *Sanguisorba officinalis* , *Styphnolobium japonicum* , *Schott*, *Prunus persica* , *Batsch*, *Sophora flavescens* , *Eupolyphaga sinensis*	Not mentioned	Herba, Seed, Root, Flos, Semen	Matrine and Oxymatrine	Aqueous Extracts	1, 2, 4 g/kg	↑ Nrf2 antioxidant response blocks cellular transformation, ↓ proliferation, and anchorage‐independent growth of HT‐29 cells	Fang et al. (2018)
Multiple classes of compounds								
8.	*Artemisia ordosica Krasch*	Not mentioned	Plant	Flavonoids, Phenolic Acids, Alkaloids, Terpenes, Amino Acids	Aqueous Extract	0.5, 2, 4, 6, 8, 10 mg/mL	↑ The colon length, ↑ antioxidant enzyme activity, ↓ inflammatory cell infiltration, ↓ TNF‐α and IL‐6, ↓ malondialdehyde (MDA) levels	Jiang et al. (2025)
9.	*Cornus mas L*.	Cornelian Cherry	Fruits	Iridoids and Polyphenols	Ethanolic Extract	20, 100 mg/kg	Counteracted the TNBS‐induced ↓ IL‐6, RORγt, and p‐STAT3 levels and a ↓ in Foxp3 level and PIAS3 mRNA expression	Szandruk‐Bender et al. (2023)
10.	*Origanum majorana L*.	Marjoram	Flowers	Betulin, Astaxanthin, Tridecanol, Bicyclo, Retinoic Acid	Ethanolic Extract	500 mg/kg/day	↓ NFκB, TNFα, IL‐1β, IL‐6, IL‐23, IL‐17, COX‐2, ↓ JAK2/STAT3 pathways, ↓Th17 cell response, ↑ the antioxidant activity, ↓ cytokine production by downregulating NFκB	Taha et al. (2023)
11.	*Manilkara zapota*	Sapodilla	Leaves	M‐Coumaric, Quinic Acid, C16 Sphinganine, 6′‐O‐Cafeate Isoorientin, And Apocynin	Aqueous Extract	50, 100, 200 mg/kg	↓ dysplasia development, ↓ ROS, superoxide production, and inflammation through the ↓ TNF‐α and IL‐6 proteins. Improved DAI scores, colon length, colon histological dysplasia and inflammation scores, and SOD level	Tamsir et al. (2024)
In vitro and in vivo								
Polyphenols								
12.	*Artemisia argyi*	Wormwood and Chinese Mugwort	Plant	Polyphenols, Phenolic Acids, and Flavonoids such as Flavones, Flavonols, and Anisoflavone Glucoside	Ethanolic Extract	10, 20, 40 μg/mL (in vitro), 200 mg/kg p.o. (in vivo)	Activation of anti‐oxidative‐associated proteins, ↓ nuclear factor‐κB (NF‐κB), ↓ NO production, the level of PGE2, and Cox‐2	Shin et al. (2022)
13.	*Aralia elata*	Angelica Tree	Plant	Polyphenol and Flavonoids	Aqueous Extract	25, 50, 75, 100 μg/mL (in vitro), 250, 500, 1000 mg/kg (in vivo)	↓ levels of DAI, NO, TNF‐α, IL‐1β, and IL‐6, ↓ inflammatory mediators in RAW 264.7 cells	Lee et al. (2022)
14.	*Centella asiatica*	Tiger Grass or Gotu Kola	Plant	Phenolic Compounds (Neochlorogenic Acid, Cryptochlorogenic Acid, 1,5‐Dicaffeoylquinic Acid), and Flavonoid (Miquelianin)	Aqueous Extract	25, 50, 100 mg/kg	↑ Levels of the anti‐inflammatory cytokines IL‐4 and IL‐10	Shin et al. (2025)
15.	*Lonicera japonica*	Japanese Honeysuckle	Buds	Chlorogenic Acid	Ethanolic Extract	200 μg/mL (in vitro), 200 mg/kg/day (in vivo)	↑ *Lactobacillus* and fecal short‐chain fatty acids (SCFAs) production alleviated inflammation, intestinal barrier injury, and oxidative stress induced by DSS	Chen et al. (2024)
Flavonoids								
16.	*Filipendula glaberrima*	Korean Meadowsweet	Plant	Flavonoids (Catechin, Miquelianin, Quercitrin, and Afzelin)	Aqueous Extract	100–400 μg/mL (in vitro), 1–25 mg/kg (in vivo)	↓ Inflammation via the MAPK and NF‐κB pathways, ↓ the phosphorylation of the mitogen‐activated protein kinase (MAPK) and nuclear factor‐κB (NF‐κB) signaling pathway‐related proteins	Jung et al. (2023)
17.	Green Tea/ *Camellia sinensis*	Tea Plant	Leaves	Catechin and Epigallocatechin‐3‐Gallate, Epigallocatechin, Epicatechin, Gallocatechin Gallate, Epigallocatechin Gallate, Epicatechin Gallate	Ethanolic Extract	40, 60 mg/mL (in vitro), 500, 1000 mg/kg oral (in vivo)	↓ expression of annexin A1 in GTE‐treated jejunum tissue, ↓ inflammation‐related signal expression, including TNFA, COX‐2, and iNOS. ↑ proliferation‐related signals such as PCNA, CD44, and Ki‐67	Kim et al. (2021)
18.	*Acanthopanax senticosus*	Not mentioned	Not mentioned	Flavonoids	Ethanolic Extract	30, 60, 120 mg/L (in vitro), 50, 100, 200 g/L (in vivo)	↓ the level of MDA, ↑ levels of SOD, CAT, and GPx	Su et al. (2022)
Multiple classes of compounds								
19.	*Bamboo shoot* and *Artemisia capillaris*	Bamboo Sprouts and Capillary Wormwood	Not mentioned	Flavonoids, Phenolic Acids, Terpenoids, Coumarins, Chlorogenic Acid, Ascorbic Acid, Scoparone, And Essential Oil	Extract	200 μg/mL for 24 h (in vitro), 250 mg/kg/day oral (in vivo)	↓ ROS generation, F‐Actin formation, and RhoA activity	Kim et al. (2022)
20.	*Alnus japonica*	Japanese Alder	Bark	Diarylheptanoids, Triterpenoids, and Flavonoids	Ethanolic Extract	30, 60 μg/mL (in vitro), 30, 60 mg/kg (in vivo)	↓ interleukin (IL)‐1β, IL‐6, tumor necrosis factor (TNF)‐α, and cyclooxygenase (COX)‐2, ↑ of tight junction proteins, zonula occludens (ZO)‐1 and occludin	Chi et al. (2018)
The class of the compound is not mentioned								
21.	*Gleditsia sinensis*	Chinese Honey Locust	Not mentioned	Not mentioned	Methanolic Extract	100 mg/mL (in vitro), 1 g/kg (in vivo)	↓ pro‐inflammatory cytokines including IL‐1β, TNF‐α, and IFN‐γ, p65 intracellular transport	Kim et al. (2015)
Ulcerative colitis								
In vivo								
Polyphenols								
22.	*Crataegus azarolus*	Hawthorn	Fruits	Polyphenols, Flavonoids, Tannins	Aqueous Extract	100, 200, 400 mg/kg	↑ H_2_O_2_ and MDA contents, ↓ PSA and enzymatic antioxidant activities (SOD, CAT, and GPx), non‐enzymatic antioxidant levels (GSH and –SH groups)	Sammari et al. (2024)
23.	*Forsythia suspensa*	Qingqiao, Laoqiao	Fruits	Polyphenol (Homovanillic Acid, Hydroquinone, Isoproterenol, Norepinephrine, P‐Synephrine, Sinapyl Alcohol and Tyrosol)	Ethanolic Extract	0.1, 0.2, 0.4 g/mL	↓ The protein levels of ASC, caspase‐1, IL‐1β, GSDMD and NLRP3	Chao et al. (2022)
24.	*Vaccinium vitis‐idaea L*.	Lingonberry	Fruits	Anthocyanin, Ellagitannin, Phenolic Acid, Polyphenols	Ethanolic Extract	10, 100 mg/kg orally	↓ MPO activity and COX‐2 and PGE2 expression in colon tissues, ↓ inflammatory cytokines (IL‐1β, IL‐6, TNF‐α, and IFN‐γ), and NO production suppressed the histological injury and inflammatory mediators (NF‐κB, iNOS)	Jeon et al. (2021)
25.	*Rubus tereticaulis*	Not mentioned	Leaves	Quinic Acid, 5‐Caffeoylquinic Acid, Quercetin Pentoside, Quercetin Glucoside, Quercetin3‐O‐Β‐D‐Glucuronide, Kaempferol‐3‐O‐Β‐D‐Glucuronide, and Kaempferol Rutinoside	Ethanolic Extract	300 mg/kg (in vivo)	Antioxidant and anti‐inflammatory mechanisms (anti‐LOX activity)	Şen et al. (2023)
26.	*Vaccinium corymbosum*	Blueberry	Fruits	Polyphenols, Flavonoids	Aqueous Extract	50 mg/kg	↓ The expression of COX‐2 and IL‐1β, the activation of NF‐κB, p65 NF‐κB, IFN‐γ, iNOS	Pervin et al. (2016)
27.	*Quercus brantii*	Brant's Oak	Fruits	Phenolic Acids and Flavonoids	Ethanolic Extract	200, 400 mg/kg	↓ TNF‐α, IL‐6, NO, MPO activity, MDA content, ↑ superoxide dismutase (SOD) activity, ↓ and body weight loss	Naini, Mehrvarzi, et al. (2021)
28.	*Aloe barbadensis* Miller	*Aloe Vera*	Leaves	Phenolic Compounds	Ethanolic Extract	200, 400 mg/kg	↓ TNF‐α, IL‐6, NO, MDA, MPO, ↑ body weight and colon weight/length ratios	Naini, Zargari‐Samadnejad, et al. (2021)
Flavonoids								
29.	*Laurus nobilis*	Laurel	Leaves	Flavonoids	Ethanolic Extract	100, 200 mg/kg	↑ Superoxide dismutase SOD, catalase CAT, and glutathione GSH, ↓ levels of thiobarbituric acid reactive substances TBARS in extract‐treated groups	Abdullah Al‐Omran et al. (2023)
30.	*Cyrtocarpa procera*	Chupandilla	Bark	Quercetin, Catechin, Kaempferol, Naringenin, Chrysin	Methanolic Extract	200 mg/kg	↓ Levels of pro‐inflammatory cytokines such as TNF‐α, IL‐1β, and IFNγ in serum, as well as the MPO activity, ↓ oxidative stress, and preventing the deterioration of the activity of antioxidant enzymes, such as SOD, CAT, and GPx, ↓lipid peroxidation damage	Rodriguez‐Canales et al. (2020)
Terpenes								
31.	*Lindera aggregata*	Evergreen Lindera	Root	Isolinderalactone, Linderane and Lindenenol	Ethanolic Extract	0.5, 1, 2 g/kg	↓ Production and secretion of IL‐6, the signal transduction of IL‐6/STAT3 signaling pathway, and modulate the balance of Th17 and Treg cells. ↓ TNF‐α, IFN‐γ, IL‐17A, and IL‐10	Lai et al. (2021)
32.	*Syringa oblata* Lindl.	Early Blooming Lilac	Leaves	Iridoid Glycosides	Hydroethanolic Extract	20, 40, 80 mg/kg	↓ TLR‐2/4/MyD88/NF‐κB Signaling Pathway. ↓ 8‐OHdG, NOX1, and NOX2 and the expression of pro‐inflammatory cytokines, such as IL‐2, IL‐4, IL‐5, IL‐12p40, and IL‐13, the protein and mRNA levels of TLR‐2, TLR‐4, MyD88, and NF‐κBp65	Zhang et al. (2020)
Multiple classes of compounds								
33.	*Caulerpa mexicana*	Green Seaweed	Green Algae	Polysaccharides, Terpenes, and Flavonoids	Methanolic Extract	2 mg/kg/day, intravenous	↓ IFN‐g, IL‐12, TNF‐α (Th1 pattern), IL‐6, and IL‐17A	Bitencourt et al. (2015)
34.	*Bletilla striata*	Hyacinth Orchid	Plant	2,7‐Dihydroxy‐4‐Methoxyphenanthrene‐2,7‐O‐Diglucoside, 3,7‐Dihydroxy2,4‐Dimethoxyphenanthrene‐3‐O‐Glucoside, Bletlol A, and Blespirol	Aqueous Extract	1, 2, 4 g/kg	↑ Factor receptor (EGFR) and PIK3CA. ↓ the secretion of the inflammatory cytokines IL‐6, TNF‐α	Gong, Lv, et al. (2023)
35.	*Thymus longicaulis* subsp. *chaubardii*	Not mentioned	Aerial Parts	Favonoids, Terpenes, Phenolic Derivatives	Ethanolic Extract	200 mg/kg/orally/day, for 3 days	Modulating pro‐ and anti‐inflammatory cytokines, the mitochondrial apoptotic pathway	Ozbeyli et al. (2025)
36.	*Rosmarinus officinalis*	Not mentioned	Aerial Parts	Flavonoids, Phenolics and Terpenoids	Methanolic Extract	120 mg/mL	Antioxidant activity	Yilmaz et al. (2022)
The class of the compound is not mentioned								
37.	*Laurus nobilis*	Bay Tree, True Laurel, Sweet Bay, Grecian Laurel Or Bay Tree	Leaves	Not mentioned	Aqueous Extract	200 μL (in vivo) p.o.	↓ Nuclear factor‐κB activation	Correa and Orlando (2023)
In vitro and in vivo								
Polyphenols								
38.	*Dilodendron bipinnatum*	Not mentioned	Inner Stem Bark	Catechin, Gallic Acid, Epigallocatechin Gallate and Gallocatechin	Hydroethanolic Extract	1, 5, 20 μg/mL (in vitro), 20, 100, 500 mg/kg (in vivo)	↓ TNF‐α and IL‐1β concentrations, IL‐17 and COX‐2 expressions, ↑ mucus production, ↓ and leukocyte migration	Oliveira et al. (2021)
39.	*Scutellaria baicalensis*	Huangqin	Root	Polyphenols	Ethanolic Extract	0.05, 0.1, 0.2, 0.4, 0.6, 0.8, 1 mg/mL (in vitro), 50, 100, 200 mg/kg (in vivo)	↑ The activity of GSH‐PX, CAT, and SOD (in vitro). ↓ MDA, DAI, MPO activity, TGF‐β1 protein expression, ↑ the activity of glutathione peroxidase, catalase, and superoxide dismutase	Jin et al. (2016)
40.	*Cotoneaster integerrimus*	Dağ Muşmulası Or Tavşan Elması	Twigs and Fruits	Protocatechuic Acid, Ferulic Acid, Hesperidin, Eriodictyol, and Apigenin	Methanolic and Aqueous Extracts	10–1000 μg/mL (in vitro), 0.1–20 mg/mL (in vivo)	↓ (H_2_O_2_) induced TNFα gene expression in HCT116	Zengin et al. (2019)
41.	*Mangifera indica* and *Punica granatum*	Mango and Pomegranate	Fruits	Polyphenols, Gallotannins, Ellagitannins, Flavonols, Quercetin, Monogalloyl Glucoside, Punicalins, Gallic Acid, Punicalagins, Oh‐Benzoic Acid Hexoside, Ellagic Acid, Dihydrophaseic Acid, Chlorogenic Acid, Gallotannins	Aqueous Extract	Ad libitum	Mango ↓ the ratio of phosphorylated/total protein expression of the IGF‐1R‐AKT/mTOR axis, mRNA expression of Igf1, Insr, and pik3cv. Pomegranate ↓ p70S6K and RPS6, as well as Rps6ka2, Map2k2, and Mapk1 mRNA. The level of IL‐1 and IL‐6	Kim et al. (2016)
Flavonoids								
44.	*Morus alba*	Mulberry	Leaves	Flavonoids (Quercetin, Kaempferol)	Ethnolic Extracts	5, 50, 150, 250 μg/mL (in vitro), 30% (in vivo)	↓ NO, prostaglandin E2 (PGE2), inducible nitric oxide synthase (iNOS), cyclooxygenase‐2 (COX‐2), and inflammatory cytokines, the reactive oxygen species (ROS) production and the scavenging of 2,2‐diphenyl‐1‐picrylhydrazyl (DPPH) free radicals	Lin et al. (2022)
45.	*Ephedra sinica*	Chinese Ephedra	Roots	Proanthocyanidins	Ethanolic And Ethyl Acetate Extract	5–320 μg/mL (in vitro), 400, 800 mg/kg (in vivo)	↓ NO production in LPS‐induced RAW 264.7, inflammatory cytokines, and regulating oxidative stress	Lv et al. (2022)
Terpenes								
46.	*Taraxacum officinale*	Dandelion	Plant	Taraxasterol Acetate	Ethanolic Extract	0–40 μM (in vitro), 25, 50, 100 mg/kg (in vivo)	↓ Secretion of pro‐inflammatory cytokines such as TNF‐α, IL‐6, and IL‐1β	Jin et al. (2024)
Multiple classes of compounds								
47.	*Rosa roxburghii* Tratt	Burr Rose	Fruits	Polyphenols, Flavonoids and Terpenoids	Ethanolic Extract	200–500 μg/mL (in vitro), 300, 500 mg/kg (in vivo)	Modulation of inflammatory responses, such as the IL‐17 and TNF signaling pathways	Li et al. (2023)
48.	*Thuja occidentalis*	Thuja, White Cedar or Tree of Life	Leaves	Tannins, Flavonoids, Polysaccharides, Ethereal Oil	Ethanolic Extract	5, 25, 50, 100 μg/mL (in vitro), 5, 25, 50 mg/kg (in vivo)	↑ Oxidative stress and attenuation of lipid peroxidation. ↓ inflammatory infiltrate, ↑ in mucosal secretion, ↓ the production of IL‐6 and TNF‐α	Stan et al. (2019)
49.	*Alstonia boonei*	Not mentioned	Stem Bark	Tannins, Saponins, Alkaloids, Steroids, Flavonoids and Phenols	Aqueous and Methanolic Extracts	50 μL (in vitro), 125, 250 mg/kg (in vivo)	↓ TNF‐α, IL‐6, IL‐1β, and PGE2 production, MDA, NO, ↑ superoxide dismutase and catalase and ↓ glutathione	Adjouzem et al. (2020)
The class of the compound is not mentioned								
50.	*Ilex rotunda* Thunb.	Kurogane Holly	Plant	10 Bioactive Components	Aqueous Extract	120 μg/mL (in vitro), 1.8 g/kg (in vivo)	Restoring the intestinal mucosal barrier and modulating the cytokine‐cytokine interaction pathways, ↓ the protein expression of oncostatin M and oncostatin M receptor in Caco‐2 cells	Li et al. (2024)
Acute and chronic colitis								
In vitro and in vivo								
Flavonoids								
51.	*Alpinia officinarum hance*	Lesser Galangal	Root	Galangin	Hexane Extract	0.5, 1, 2.5, 5, 7.5, 10 μg (in vitro), 200 mg/kg (in vivo)	↓ Proliferation of HT‐29 cells, mRNA expression of NF‐B and COX‐2, AST, ALT, ALP, LDH and ‐GT, expressions of the pro‐inflammatory mediators NF‐B and TNF‐ i. NF‐kB, and COX‐2	Rajendiran et al. (2018)
Multiple classes of compounds								
52.	*Morinda officinalis*	Indian Mulberry	Plant and Root	Iridoid and Anthraquinones	Alcoholic Extract	50, 100, 200 μg/mL (in vitro), 20, 40, 80 mg/kg/day (in vivo)	↓ Serum levels of pro‐inflammatory cytokines TNF‐α, IL‐6, and IL‐17	Liang et al. (2017)
Crohn's disease and ulcerative colitis								
In vivo								
Polyphenols								
53.	*Brassica oleracea var Capitata rubra*	Red Cabbage	Leaves	Polyphenols, Anthocyanins	Ethanolic Extract	5 mg/kg twice daily (the final volume of 150 μL)	↓ Pro‐inflammatory cytokine release	Zielińska et al. (2015)
Colitis and colon cancer								
In vitro and in vivo								
Polyphenols								
54.	*Allium sativum*	Garlic (Nubia Red Garlic)	Garlic Cloves	Catechin, Fenols, Gallic Acid, Chlorogenic Acid, P‐Coumaric Acid, T‐Ferulic Acid, Benzoic Acid, Resveratrol, Naringenin, Hesperetin, and Flavone	Hydroalcoholic Extract and Aqueous Extract	1, 10, 100 μg/mL (in vitro)	↓ LPS‐induced TNF‐α, NF‐κB, and IL‐6 gene expression, prostaglandin (PG)E2, 8‐iso‐PGF2α, ↑ the 5‐hydroxyindoleacetic acid/serotonin ratio	Recinella et al. (2022)
Flavonoids								
55.	*Tetrastigma hemsleyani*	Not mentioned	Radix	Flavones	Not mentioned	1.6, 3.2, 6.4 mg/mL (in vitro), 30, 60 mg/kg (in vivo)	Induce cell cycle arrest at G0/G1 phase and ↓ epithelial‐mesenchymal transition process, ↓ β‐catenin activation, and protein expression	Wu et al. (2018)

Abbreviations: ↑, increased/upregulated; ↓, decreased/downregulated.

Across the studies, plant extracts consistently reduced intestinal inflammation and oxidative stress. Most of them downregulated pro‐inflammatory cytokines (TNF‐α, IL‐6, and IL‐1β) and enhanced antioxidant enzyme activity (SOD, CAT, GPx), supporting their role in restoring intestinal homeostasis. Extracts from *Lycium ruthenicum*, *Artemisia argyi*, *Lindera aggregata*, and *Achyrocline satureioides* showed notable efficacy by improving epithelial integrity through increased expression of tight junction proteins (ZO‐1, occludin, claudin‐1).

Table 2 compiles data from more than 50 experimental studies investigating 71 plant species for their potential anticancer activity against colon carcinoma using both in vitro and in vivo models. The summarized plants belong to diverse taxonomic groups and include herbs, fruits, roots, and marine species, all rich in bioactive compounds, such as polyphenols, flavonoids, terpenes, alkaloids, and polysaccharides. Extracts were typically obtained using ethanol, methanol, aqueous, or mixed solvents and were tested at doses ranging from microgram to gram levels, depending on the model system.

**TABLE 2:** Bioactive compounds tested against colon cancer.

Nr.	Plant name	Common name	Part used	Most important bioactive components	Administration/Type of Extract	Dose concentration	Mechanism	References
Colon cancer								
In vitro								
Polyphenols								
1.	*Hypericum triquetrifolium*	Curled‐Leaved St. John's‐Wort	Plant	Phenols	Hydroethanolic Extract	0.064, 0.125, 0.25, 0.5 mg/mL	Induced cell death via an apoptotic process	Mahajna et al. (2019)
2.	*Persea americana*	Avocado	Seeds	Polyphenol	Methanolic Extract	19–132 μg/mL	↓ Lipid hydroperoxide formation, nuclear translocation of nuclear factor κB	Dabas et al. (2019)
3.	*Urtica dioica*	Common Nettle, Burn Nettle	Leaves	Phenols	Aqueous Extract	1, 2, 4 mg/mL	Antibacterial and antioxidant properties	Dakhli et al. (2025)
4.	*Euphorbia lathyris*	Caper Spurge	Seed	Polyphenols, Including Esculetin, Euphorbetin, Gaultherin and Kaempferol‐3‐Rutinoside	Ethanolic Extract	IC50 T‐84 (16.3 ± 2.54 μg/mL) HCT‐15 (IC50 of 72.9 ± 1.27 μg/mL)	Cell death was mediated by the overexpression of caspases 9, 3, and 8, and by activation of autophagy	Mesas et al. (2021)
5.	*Moringa oleifera* , *Tropaeolum tuberosum* and *Annona cherimola*	Drumstick Tree, Mashua And Cherimoya	Seed, Seed, Tubers	Polyphenols	Ethanolic Extracts	2.5, 5, 10, 20, 40 μg/mL	Overexpression of caspases 9, 8 and 3, ↑ production of reactive oxygen species, ↓G2/M phase cells	Fuel et al. (2021)
6.	*Aronia*	Chokeberries	Leaves	Phenolic Acids, Flavonols and Anthocyanin Pigment	Methanolic Extracts	50, 100 μg/mL	↓ The migration and invasion of SW‐480 cells, the product of MMP‐2 and MMP‐9 gene expression, ↓ and the activity of both MMPs	Owczarek et al. (2024)
7.	*Glycine max*	Soybean	Leaves	Polyphenol and Flavonoid	Ethanolic Extract	0, 62.5, 125, 250, 500 μg/mL	↓ Migration and adhesion, the production of NO, and PGE2	Kwak and Ju (2017)
8.	*Ribes taxa*	Blackcurrants	Seeds	Hydroxylated Benzoic Acids, Cinnamic Acid Derivatives, Flavonoids, Flavonoid Glycosides, Flavonol Derivatives (Populnin, Quercetin, Isoquercitrin, Rutin, Kaempferol, Quercitrin, Myricetin, Fisetin, Astragalin, Nicotiflorin, And Galangin. Fisetin, Luteolin, Eriodictyol, Phloretin, Galangin, and Naringenin)	Methanolic Extract	0–300 μg/mL	Not mentioned	Lyashenko et al. (2024)
9.	*Ribes nigrum L*, *Vacciniummyrtillus L*., *Vaccinium vitis‐idaea L*.	Blackcurrant, Bilberry and Lingonberry	Young Shoots	Polyphenols, Flavonoids and Condensed Tannins	Ethanolic Extract	20–800 μg/mL	Antioxidant activity	Solcan et al. (2023)
10.	*Ribes nigrum*	Blackcurrant	Fruits	Polyphenolic Compounds, Including Anthocyanins, Flavan‐3‐Ols, Hydroxycinnamic Acid Derivatives, and Flavonols. Group Of Flavan‐3‐Ols (Epicatechin, Gallocatechin and Epigallocatechin), Hydroxycinnamic Acids (Derivatives Of Caffeic, Ferulic, Sinapic and P‐Coumaric Acids) and Flavonols (3‐Oglucosides and 3‐O‐Rutinosides Of Myricetin, Quercetin, Kaempferol and Isorhamnetin)	Aqueous Extract	0.01–10 mg/mL	↓ Of oxidative‐stress‐mediated MMP expression via the redox‐sensitive NF‐κB pathway, ↓ cell invasion by ↓ matrix metalloproteinase MMP‐2 and MMP‐9 expression	Olejnik et al. (2018)
11.	*Rubus caesius*	Dewberry	Leaves	Polyphenols (Ellagic Acid, Sanguiin H‐6, and Flavonol Derivatives), Quercetin and Kaempferol, as well as Ellagitannins, Flavonoids	6 Extracts	50, 100, 150, 200, 250 μg/mL	Not mentioned	Grochowski et al. (2016)
12.	*Aristotelia chilensis*	Maqui Berry	Fruits	Polyphenols, Anthocyanins, Flavonoids	Ethyl Acetate, Acetone, Aqueous and Methanolic Extract	1, 5, 10, 15, 30, 50 μg/mL	↓ Production of nitric oxide (NO), nitric oxide synthases (iNOS), NF‐B and COX‐2 protein expressions and a potent antioxidant activity, and cell growth	Céspedes‐Acuña et al. (2018)
13.	*Morus nigra*	Black Mulberry	Fruits	Phenol, Flavonoid, Anthocyanin, Gallic Acid, and Quercetin	Aqueous and Ethanolic Extracts	10, 20, 40, 80 mg/L	↓ Cell growth, ↓ intracellular ROS, intracellular Ca^2+^ level, ↓ MMP loss	Cui et al. (2020)
14.	*Vaccinium meridionale* Swartz	Andean Berry	Fruits	Phenols and Anthocyanins	Aqueous Extract	10%, 20%, 30%, 40% v/v	Triggered apoptosis through the cell cycle arrest in phases S, G2/M and SubG0/G1, without promoting the mitochondrial membrane damage and oxidative stress, suggesting the activation of the intrinsic apoptotic pathway	Arango‐Varela et al. (2021)
15.	*Curcuma longa*	Turmeric	Not mentioned	Curcumin (Diferuloylmethane)	Aqueous Extract	Not mentioned	↓ Adenosine deaminase activity	Durak et al. (2015a)
16.	*Morchella sextelata*	Not mentioned	Fruiting Bodies	Polyphenols	Ethanolic Extract	2, 4, 6, 8, 10, 12 and 14 mg/mL (in vitro)	↓ Expression of Bcl‐2, ↑ the expression of Bax, caspase‐3, and caspase‐9	Zhai et al. (2025)
Flavonoids								
17.	*Viburnum opulus*	Guelder Rose	Fruits	Flavonoids	Methanolic Extract	5–2000 μg/mL (in vitro)	↑ Intracellular ROS, cytotoxicity, DNA damage, and apoptosis	Bozali et al. (2020)
18.	*Origanum majorana*	Marjoram	Leaves	Luteolin, Quercetin	Ethanolic Extract	75, 150, 300, 450, 600 μg/mL	Triggered abortive autophagy and activated a caspase 3 and 7‐dependent extrinsic apoptotic pathway, ↑ cell cycle regulator p21, lipidized LC3II, a marker of autophagy	Benhalilou et al. (2019)
19.	*Sambucus nigra* , *Vaccinium myrtillus* , *Rubus fruticosus* , *Ribes nigrum*	Elderberry, Bilberry, Blackberry and Black Currant	Fruits (Juice)	Anthocyanin	Ethanolic Extract and Dimethyl Sulfoxide Extracts	6.25–200 μg/mL	To induce the NF‐κB pathway and, the transcription of pro‐inflammatory cytokines IL‐1β, IL‐8, TNF‐α and COX‐2	Schmutz et al. (2024)
Terpenes								
20.	*Ferula hermonis*	Not mentioned	Root	3,5‐Dimethylbenzenemethanol, Alpha‐Pinene, Alpha‐Bisabolol, and Baccatin III, Α‐Curcumene	Hexane Extract	0–100 μg/mL (in vitro), 100 mg/kg (in vivo)	Apoptotic pathway	Abutaha et al. (2019)
21.	*Origanum majorana*	Marjoram	Not mentioned	Terpinen‐4‐Ol, Alpha‐Terpinol, Alpha‐Pinene, Camphene, P‐Cymol, B‐Caryophyllene, Bicyclogermacrene and Neophytadiene	Essential Oil	64, 128, 256, 640 μg/mL	Triggers p38 MAPK‐mediated protective autophagy, apoptosis, and caspase‐dependent cleavage of P70S6K	Athamneh et al. (2020)
Multiple classes of compounds								
22.	*Aronia melanocarpa* , *Cornus mas* , and *Chaenomeles superba*	Black Chokeberry, Cornelian Cherry, Flowering Quince	Leaves	Polyphenols, Flavonols, Saponins, Alkaloids, Steroids, Glycosides, Carbohydrates, Proteins, Quinones, and Coumarin	Aqueous Extract	0.01%, 0.02%, 0.04%, 0.08%, 0.16%, and 0.63%	Partial detachment of cells, necrotic cells, chromatin condensation, cytoplasmic vacuolization, cell nuclei lysis, and nucleus fragmentation	Efenberger‐Szmechtyk et al. (2020)
23.	*Prunella vulgaris* L.	Heart‐Of‐The‐Earth	Spike	Triterpenes, Flavonoids, Phenolic Compounds, and Carbohydrates	Ethanolic Extract	0.25, 0.50, 0.75, 1, 1.25, 1.5 mg/mL	↓ miR‐34a rescued the expression of these target genes, ↓ growth of HCT‐8 cells by targeting Notch1, Notch2, and Bcl‐2 via activation of miR‐34a	Fang et al. (2017)
24.	*Sargassum ilicifolium*	Not mentioned	Plant (Seaweed)	Alkaloids, Steroids, Terpenoids, Fatty Acids, Saponins and Tannins	Ethanolic Extract and Chloroformic Extract	7.8, 15.6, 31.2, 62.5, 125, 250, 500, 1000 μg/mL	Anti‐proliferative activity	Sumithra et al. (2017)
25.	*Tribulus terrestris* L.	Goathead	Fruits	Flavonoids, Flavonol Glycosides, Steroidal Saponins and Alkaloids	Methanolic Extract	0.5–5 μg/mL	↑ Concentration of the extract, ↓ DNA synthesis	Pourali et al. (2016)
26.	*Innotus obliquus*	Chaga Mushroom	Fruiting Body	Polyphenols, Triterpenoids, Steroids, Ergosterol Peroxides, Inotodial, and 3β‐Hydroxy‐Lanosta‐8,24‐Dien‐21‐Al, A Lignin‐Like Substance	Ethanolic Extract	2.5–10 μg/mL	↓ Viable HT‐29 cell numbers and DNA synthesis, ↑ the percentage of cells in G1 phase, ↓ protein expression of CDK2, CDK4, and cyclin D1, ↑ expression of p21, p27, and p53, ↓ phosphorylation of Rb and E2F1 expression	Lee et al. (2015)
27.	*Annona muricata*	Soursop, Graviola	Fruits	Acetogenins, Alkaloids, Flavonoids, Tannins and Saponins Compounds	Ethyl Acetate Extract	6.25–400 μg/mL (IC50 value of 10.56 μg/mL)	↓ Cells' proliferation and cell viability, inducing apoptosis, and arresting the cell cycle at the G0/G1 phase	Daddiouaissa et al. (2021)
28.	*Malus* spp. *Cultivars* (*Ligol*, *Rubin*, and *Auksis*, *Kostele* and *Paprastasis antaninis*)	Apple	Whole Fruit and Peel	Phenolic (Quercetin Glycosides, Rutin, Hyperoside, Isoquercitrin, Reynoutrin, Avicularin and Quercitrin), Flavan‐3‐Ols (Procyanidin B1, Procyanidin B2, Procyanidin C1, (+)‐Catechin, And (−)‐Epicatechin), Dihydrochalcones (Phloridzin) and Phenolic Acids (Chlorogenic Acid) And Triterpene (Betulinic Acid, Corosolic Acid, Oleanolic Acid and Ursolic Acid) Compounds	Ethanolic Extract, Acetone Extract	(concentration 30 mg/mL) 15 μL	The apple extracts inhibited hyaluronidase	Butkeviciute et al. (2021)
29.	*Celtis aetnensis* (*Tornab*.) *Strobl*	Evergreen Hackberry	Twigs	Betulin, Gallic Acid, 3,3′‐Di‐O‐Methylellagic Acid, Moretenol and Two Triterpene Esters, 3β‐Transsinapoyloxylup‐20 (29)‐En‐28‐Ol and 3β‐Trans‐Feruloyloxy16β‐Hydroxylup‐20 (29)‐Ene and Five Known Triterpenes, 3β‐O‐(E)‐Feruloylbetulin, 3β‐O‐(E)‐Coumaroylbetulin, Betulin, 20‐Epibryonolic Acid, and Ursolic Acid	Chloroformic Extract	5, 50, 100, 250, 500 μg/mL	↑ The levels of ROS, ↓ in RSH levels and in the expression of HO‐1, ↑ in LDH release	Acquaviva et al. (2016)
30.	*Salvia officinalis*	Sage	Aerial part of the plant	Oxygenated Monoterpenes, Α‐Thujone, 1,8‐Cineole (Eucalyptol) and Camphor	Essential Oil	20, 50, 100, 200 μg/mL	↑ G0/G1 phase	Luca et al. (2020)
31.	7 *Cornus mas L*.	Cornelian Cherry	Fruits	Polyphenols and Iridoids, Loganic Acid, Cornuside, Cyanidin 3‐Galactoside, and Pelargonidin 3‐Galactoside, Anthocyanins	Hydroethanolic Extract	9.14–13.97 mg/mL	Inhibitory activity toward carbohydrate digestive enzymes α‐amylase and α‐glucosidase	Blagojević et al. (2021)
32.	*Acmella caulirhiza*	Not mentioned	Leaves and Flowers	Alkaloids, Flavonoids, Terpenoids, Polyphenolic Compounds, and Tannins	Aqueous, Hydroethanolic and Ethanolic Extract	5, 10, 50, 100, 250, 500 μg/mL	↓ Proliferation of Cancer Cells	Tienoue Fotso et al. (2024)
Other class of compounds								
33.	*Persea americana var. drymifolia*	Mexican Avocado	Seed	Lipid‐Rich	Hexane Extract	20, 50, 75, 100, 150 μg/mL	↑ Apoptosis, ↓membrane mitochondrial potential, fatty acid oxidation, ↑ superoxide production and mitochondrial ROS, IL‐6, IL‐8 and IL‐10, ↓ IL‐1β secretion	Lara‐Márquez et al. (2020)
34.	*Rheum rhabarbarum*	Rhubarb	Plant	Anthraquinones	Aqueous Extracts	Not mentioned	↓ Adenosine Deaminase activity	Durak et al. (2015b)
In vivo								
Polyphenols								
35.	*Punica granatum*	Pomegranate	Peel	Polyphenols	Methanolic Extracts	2.25, 4.5 g/kg, p.o.	↓ Wnt/‐Catenin, ↑ apoptosis, and mitigates inflammation↓, tumor cell proliferation in vivo	Ahmed et al. (2017)
Polysaccharides								
36.	*Crataegus pinnatifida*	Hawthorn	Plant	Polysaccharides	Aqueous Extract	50, 100, 200 mg/kg	↓ The inflammation, ↑ cancer cell apoptosis, ↑ expression of caspase‐3 signaling pathway, ↓ level of colonic IL‐6	Ma et al. (2023)
In vitro and in vivo								
Polyphenols								
37.	*Origanum majorana*	Marjoram	Plant	Polyphenols and Flavonoids	Aqueous, Petroleum Ether, Dichloromethane, Ethyl Acetate, Methanolic Extracts	50–550 μg/mL (in vitro), 5, 10 g/kg (in vivo)	Not mentioned	Makrane et al. (2018)
38.	*Phlomis fruticosa* and *Phlomis herba*	Not mentioned	Stems, Leaves and Flowers	Phenol (Rutin, Benzoic Acid, Epicatechin) and Flavonoid	Aqueous, Hydroalcoholic, Ethanolic Extracts	10–1.000 μg/mL (in vitro), 100 μg/mL (ex vivo)	↓ Nitrite, MDA, and 5‐HT levels, ↑ levels of LDH	Ferrante et al. (2019)
Multiple classes of compounds								
39.	*Strobilanthes crispus*	Pecah Beling, Pokok Pecah, Pecah Kaca, or Jin Batu	Plant	Phenolic Compounds (Verbascoside, Glycosidic Ester of Caffeic Acid) And Phenolic Acids (P‐Hydroxy Benzoic, P‐Coumaric, Caffeic, Vanilic, Gentinic, Ferulic and Syringic Acid), Flavonoid Components (Catechins), Caffeine, and Tannin	Ethanolic Extract	250, 500 mg/kg (5 mL/kg)	↓ Malonaldehyde (MDA) and lactate dehydrogenase (LDH), ↑ SOD, APC, Bax and Slc24a3, ↓ of Defa24 and Bcl‐2	Al‐Henhena et al. (2015)
40.	*Annona reticulata*	Bullock's Heart, Custard Apple, Netted Custard Apple, Wild‐Sweetsop and ‘Ramphal’	Leaves	Saponins, Flavonoids, Terpenoids, Tannins, Quinine, Carbohydrates and Steroids	Aqueous Extract, Alcoholic Extract	0.55 mg/mL alcoholic extract and > 7.25 mg/mL aqueous extract (in vitro), 5, 10 mg/kg, p.o. (in vivo)	↑ RBC, hemoglobin and platelet levels, endogenous antioxidant enzymes—CAT, SOD, and GSH, ↓ MDA levels and the lipid peroxidase activity, inflammation and repair the colon's damaged muscle layers	Khan et al. (2021)
Xenograft (in vitro + in vivo)								
Polyphenols								
41.	*Prunus spinosa*	Blackthorn	Drupes	Flavonoid, Phenolic Acid, and Anthocyanins	Alcoholic Extract	1, 2, 5, 10 mg/mL (in vitro), 0.05, 0.15 mg/mL (in vivo)	↓ Growth and colony formation of colon carcinoma cells (HCT116)	Condello et al. (2019)
42.	*Ecklonia cava*	Paddle Weed	Plant	Polyphenols	Aqueous Extract	250, 500, 1000 mg/kg (in vitro)	↓ Volume and weight of CT26 tumors, ↑ level of apoptotic proteins, ↓ cell proliferation, metastasis ability, ↑ tumor‐suppressing activity. activation of apoptosis, ↓ migration ability	Gong, Kim, et al. (2023)
43.	*Thalassia testudinum*	Turtlegrass	Leaves	Phenolic Components, Flavonoids, and Proanthocyanidins	Hydroethanolic Extract	1–1000 μg/mL (in vitro), 10, 50, 100 mg/kg (in vivo)	Triggers ATF4‐P53‐NFκB specific gene expression and autophagy stress pathways, ↓ cell growth, cell motility, and angiogenesis in vitro and promotes antitumor immunogenic cell death in vivo	Hernández‐Balmaseda et al. (2021)
44.	*Sasa quelpaertensis*	Bamboo Grass	Leaves	Tricin and P‐Coumaric Acid	Ethanolic Extract	100, 200, 300 μg/mL (in vitro), 300 mg/kg (in vivo)	↓ β‐catenin and phosphorylated GSK3β, ↓ tumorigenesis and the expression of CSC markers such as CD133, CD44, DLK1, Notch1, and Sox‐2, and by regulating components of the Wnt/β‐catenin and hypoxia related HIF‐1α signaling pathways	Min et al. (2015)
45.	*Artemisia annua*	Sweet Wormwood	Plant	Phenolic Compounds	Ethanolic Extract	20, 30, 40, 60, 80, 100 μg/mL (in vitro), 20, 40 mg/kg/day (in vivo)	By modulating PTEN/p53/PDK1/Akt/signal pathways through PTEN/p53‐independent manner, ↓ PDK1, AKT, MDM2, Bcl‐2, and pro‐caspase 3, as well as activation of PTEN, p53‐upregulated modulator of apoptosis (PUMA), ↑ LDH release in cancer cells	Kim et al. (2017)
46.	*Euphorbia lathyris*	Caper Spurge	Seed	Polyphenols, Including Esculetin, Euphorbetin, Gaultherin and Kaempferol‐3‐Rutinoside	Ethanolic Extract	250 mg/kg intraperitoneal injection every 3 days for a total of 19 doses	↓ Levels of pro‐inflammatory cytokines TNF‐alpha, IL‐6 and IL‐1b, ↑ antioxidant enzymes (especially in SOD2, GPX2, and NFE2L2)	Mesas et al. (2022)
47.	*Gnetum gnemon*	Melinjo	Seed	Phenolic Compounds (Gnetin C)	Extract	1.25, 12.5, 25, 50, 100, 200, 400 μg/mL (in vitro), 50, 100 mg/kg (in vivo)	↑ Apoptosis in cancer cells via caspase‐3/7‐dependent and ‐independent mechanisms, ↓ proliferation, intratumoral angiogenesis and liver metastases in BALB/c	Narayanan et al. (2015)
48.	*Rosmarinus officinalis*	Rosemary	Plant	Polyphenols	Extract	20, 40 μg/mL (in vitro), 200 mg/kg three times a week, p.o. (in vivo)	↑ Intracellular ROS that resulted in necrotic cell death	Pérez‐Sánchez et al. (2019)
49.	*Hippophae rhamnoides*	Sea Buckthorn	Fruits	Polyphenols, Flavonoids (Kaempferol)	Ethanolic Extract	20, 40, 80 μg/L (in vitro), 50 mg/kg (in vivo)	↑ Cell cycle arrest and apoptosis of colon cancer cells	Wu et al. (2021)
50.	*Olea europaea var. Sylvestris*	Olive Tree	Leaves	Polyphenol (Oleuropein and Hydroxytyrosol)	Aqueous Extract	5, 10, 20, 30, 50, 70 μg/mL (in vitro)	↑ Caspase‐dependent apoptosis in colon cancer cells via the mitochondrial apoptotic pathway, ↓ colon carcinoma HCT116 tumor growth	Zeriouh et al. (2017)
51.	*Rhus coriaria*	Sumac	Fruits	Tannins, Phenolic Acids, Flavonoids	Ethanolic Extract	75, 150, 300, 450, 600 μg/mL (in vitro), 50 mg/kg (in vivo)	↑ Protein ubiquitination, proteasomal degradation and triggers noncanonical Beclin‐1‐independent autophagy and apoptotic cell death, induces a caspase‐7‐dependent apoptosis in colon cancer cells	Athamneh et al. (2017)
Flavonoids								
52.	*Agrimonia pilosa*	Hairy Agrimony	Roots	Flavonoids (Quercetin and Quercitrin)	Methanolic Extract	Quercetin and quercitrin were injected intraperitoneally at concentrations of 2, 10, 25, 50 μg/mL (in vitro), 0.5, 0.151, and 0.224 mg/10 g (in vivo)	↓ Migration and invasion through the JNK signaling pathway	Trinh et al. (2022)
Terpenes								
53.	*Mesua ferrea*	Ceylon Ironwood	Resin	Isoledene and Elemene	Ethanolic Extract and Hexane, Chloroform Fractions	100, 200 mg/kg	↓ Multiple pro‐survival proteins, ↑ expression of ROS, caspase‐3/7 and TRAIL‐R2 in HCT 116, induced nuclear apoptosis, chromatin condensation	Asif et al. (2019)
54.	*Taraxacum officinale*	Dandelion	Root	Α‐Amyrin, Β‐Amyrin, Lupeol and Taraxasterol	Aqueous Extract	0.5–6.0 mg/mL (in vitro), 40 mg/kg/day (in vivo)	↓ Growth of both p53 WT and p53 mutant tumors in vivo, ↑ caspase 1, KCNIP1, SNCA, TNF, ↓ pro‐survival genes, especially anti‐apoptotic genes, Bcl‐2, Bcl‐2A1, and PARP2	Ovadje et al. (2016)
Other class of compounds								
55.	*Scaphium affine*	Malva Nut Tree	Seed	Β‐Sitosterol	Ethanolic Extract	75, 125 mg/mL (in vitro), 75, 100 mg/kg/day (in vivo)	↑ Anoikis by regulating the EGFR/AKT Pathway	Kawk et al. (2021)
Multiple classes of compounds								
56.	*Astragalus mongholicus Bunge‐Curcuma aromatica Salisb*	Mongolian Milkvetch‐Wild Turmeric	Not mentioned	Flavonoids (Calycosin‐7‐Glucoside, Ononin, Calycosin, Formononetin), Astragalosides (Astragaloside A, Astragaloside II, Astragaloside I) Curcumins (Bisdemethoxycurcumin, Demethoxycurcumin, Curcumin) and Essential Oils (Curdione, Curzerene, Germacrone and Β‐Elemene)	Aqueous Extract	0.08–2.07 mg/g (in vitro), 0.32, 0.64, 1.28 g/kg/day (in vivo)	↑ Level of SCFAs such as propionic acid and butyric acid, ↓ growth and metastasis of colon cancer.	Gu et al. (2021)
57.	*Ganoderma lucidum*	Reshi, Lingzhi	Sporoderm‐Broken Spores	Triterpenoids and Polysaccharides	Ethanolic Extract	1.6–10 mg/mL (in vitro), oral gavage of 75 and 150 mg/kg (in vivo)	↑ Apoptosis and cell cycle arrest at G0/G1 phase, ↓ HCT116 cell migration via ↓ MMP‐1, MMP‐2, ↑ E‐cadherin expression at mRNA levels	Li et al. (2017)
58.	*Inula Viscosa*	False Yellowhead	Leaves	Polyphenols (Caffeoylquinic Acid and Dicaffeoylquinic Acid) and Sesquiterpens	Aqueous Extract	100–300 μg/mL (in vitro), 150, 300 mg/kg (in vivo, intra‐peritoneum)	↓ Tumor growth, cell viability, cell proliferation, and induced apoptosis of colon cancer cells	Bar‐Shalom et al. (2019)
59.	*Heterobasidion annosum*	Not mentioned	Fruiting Bodies	Indoles (5‐Hydroxy‐L‐Tryptophan, L‐Tryptophan, and 6‐Methyl‐D, L‐Tryptophan), Phenolic Acid, and Sterols	Methanolic Extract	0.1, 1, 5 μg/mL (in vivo), 175, 560, 1792, 2000 mg/kg (in vivo)	↑ Expression of NF‐κB and p53 pro	Sadowska et al. (2020)
60.	*Gnetum montanum*	Not mentioned	Stems and Rhizomes	Stilbenes, Flavonoids, Sterols and Alkaloids	Ethanolic Extract	10, 20, 40, 80, 120 μg/mL (in vitro), 28, 56 mg/kg/day (in vivo mice), 200, 400, 800 μg/mL (in vivo zebrafish)	↑ Apoptosis by ↓ activation of AKT in SW480 human colon cancer cells, ↑ expression cleaved caspase‐3, cleaved PARP, and Bax,↓ proteins P‐AKT, P‐GSK‐3b, P‐PDK1, P‐c‐Raf, caspase‐3, and Bcl‐2	Pan et al. (2022)
61.	*Heterobasidion Annosum*	Not mentioned	Fruiting Bodies	Polysaccharides, Lectins or Terpenoids	Methanolic Extract	1, 2, 5 mg/kg (in vivo)	↑ Pathways of apoptosis	Sadowska et al. (2023)
The class of the compound is not mentioned								
62.	*Hedyotis diffusa willd*	Not mentioned	Plant	Not mentioned	Ethanolic Extract	0.5, 1, 2 mg/mL (in vitro), 1 g/kg (in vivo)	↑ Cytochrome C, caspase‐3, caspase‐9 and PARP, ↓ expression of Pim‐1, a potential oncogene, Bcl‐2 expression, ↑ Bax expression, ↓ cell proliferation, ↑ apoptosis in xenograft tumors, ↓ expression of COX‐2, iNOS, eNOS and HIF‐1α, levels of phosphorylated AKT, Erk1/2, JNK, p38, p70S6K, and STAT3	Feng et al. (2017)
63.	*Portulaca oleracea*	Common Purslane	Plant	Not mentioned	Ethyl Alcohol Extract	0.07–2.25 μg/mL	↓ Nodule formation of colon cancer stem cells by ↑ gene expression of the Notch signal transduction pathway	Jin et al. (2017)
64.	*Curcuma longa*	Turmeric	Rhizome	Not mentioned	Ethanolic Extract	200 mg/kg, orally administered	↓ PDX growth with sharp ↓ of mouse body weight and only mild anti‐metastasis activities	Li et al. (2021)
65.	*Caulis Spatholobi*	Not mentioned	Not mentioned	Not mentioned	Ethyl Acetate Extract	25, 50, 100, 200, 400 μg/mL (in vitro), 200 μL (in vivo)	↓ PDGF‐B is produced by platelet degranulation, ↓ tumor metastasis	Sun et al. (2020)
66.	*Tripterygium wilfordii*	Thunder God Vine	Plant	Not mentioned	Methanolic Extract	0–500 μg/mL (in vitro)	↑ Antiproliferative effects	Zhu et al. (2018)
Carcinogenesis								
In vivo								
Multiple classes of compounds								
67.	*Moquiniastrum polymorphum ssp floccosum*	Cambará	Trunk Barks	Lactones, Sesquiterpenes, Diterpenes, Triterpenes, Flavonoids, Coumarins, Phenolic Compounds and Essential Oils	Ethnolic Extract	100 mg/kg, oral gavage	Chemopreventive and anticarcinogenic activity	Limeiras et al. (2017)
In vitro and in vivo								
Polyphenols								
70.	*Houttuynia cordata*	Fish Mint	Leaves	Gallic Acid, Protocatechuic Acid, 4‐Hydroxybenzoic Acid, Chlorogenic Acid, Vanillic Acid, Syringic Acid, P‐Coumaric Acid, Ferulic Acid, Ellagic Acid, Trans‐Cinnamic Acid, Catechin, Epicatechin, Rutin, Quercetin, Luteolin, Apigenin, Quercitrin, Myricetin, Naringenin, Kaempferol, and Isorhamnetin	Ethanolic Extract	500 μg/mL (in vitro), 500 mg/kg p.o. (in vivo)	↓ NO production in RAW264.7 macrophages	Singai et al. (2024)
71.	*Vitis* spp.	Grape	Seed	Resveratrol, Catechin and (−)‐Epicatechin Monomers	Extract	6.25, 12.5, 25 μg/mL (in vitro), 0.03, 0.12% (in vivo)	↓ Protein levels of Wnt/β‐catenin pathway, c‐Myc and cyclin D1, ↑ mitochondrial‐mediated apoptosis in colon CSCs, p53, Bax/Bcl‐2 ratio, ↓ nuclear COX‐2 levels	Reddivari et al. (2016)

Abbreviations: ↑, increased/upregulated; ↓, decreased/downregulated.

Across these studies, numerous phytochemicals demonstrated strong antiproliferative, proapoptotic, and antioxidant activities. Mechanistically, most extracts inhibited cell growth by inducing apoptosis through mitochondrial or caspase‐dependent pathways, regulating signaling cascades such as NF‐κB, JNK, Wnt/β‐catenin, and PI3K/Akt, or by halting the cell cycle in G0/G1 or G2/M phases. Compounds from *Origanum majorana* , *Rhus coriaria* , *Artemisia annua* , and *Gnetum montanum* were particularly effective, suppressing tumor proliferation and metastasis while activating tumor‐suppressor genes such as p53 and Bax.

Overall, Tables 1 and 2 provide a comprehensive list of the plants featured in the eligible articles, their main bioactive components, and their mechanisms of action.

## 3 Phytochemicals. Bioactive Compounds With Antioxidant and Anti‐Inflammatory Roles

Nutraceuticals have attracted growing scientific interest due to their broad spectrum of biological activities, including anti‐inflammatory, antioxidant, anticancer, antimicrobial, and prebiotic properties, as well as beneficial effects on lipid metabolism (Andrei et al. 2014; Rachtan‐Janicka et al. 2021). Fruits and vegetables represent the primary dietary sources of phenolic compounds (Andrei et al. 2014). Numerous studies have demonstrated that diets rich in these foods reduce the risk of IBD (Bibi et al. 2018) and various cancers (Rytsyk et al. 2020), with vegetables often providing the most pronounced protective effect (Pedro et al. 2016).

Bioactive phytochemicals support intestinal homeostasis by modulating gut microbiota composition and enhancing gut barrier integrity through their antioxidant and anti‐inflammatory mechanisms (Gu et al. 2021). Natural dietary antioxidants are increasingly recognized as a key strategy to mitigate oxidative stress. Recent evidence indicates that antioxidant‐rich diets may provide greater physiological benefits than high‐dose synthetic supplements which, in excessive amounts, can become ineffective or even harmful (Chen et al. 2024).

In light of the side effects and limited efficacy of conventional pharmacological treatments, there is a growing interest in identifying natural alternatives for managing IBD. Among these, polyphenols, terpenoids, and alkaloids have shown notable therapeutic promise (Peng et al. 2019). Phenolic compounds, in particular, can be structurally categorized into two main groups: flavonoids, comprising anthocyanidins, flavanols, flavanones, flavonols, and isoflavones, and non‐flavonoids, which include phenolic acids (such as hydroxybenzoic acids), coumarins, stilbenes, lignans, and tannins (Andrei et al. 2014).

A recent 2025 review provided an updated overview of the absorption, metabolism, and tissue distribution of dietary polyphenols, including flavonoids (Williamson 2025). While some polyphenols are absorbed in the small intestine, most pass to the colon, where they are metabolized by the gut microbiota, highlighting their potential impact on colonic health. Once absorbed, these compounds circulate primarily as conjugated metabolites and can reach organs, such as the liver, kidneys, heart, and brain, depending on their lipophilicity and transporter specificity. The liver serves as the main site for conjugation, after which metabolites are either released into plasma or eliminated via bile or urine. Free (unconjugated) polyphenols are absorbed more readily, whereas conjugated forms may require hydrolysis in the intestine, emphasizing the influence of individual gut microbiota. Polyphenols attached to sugars or acids, such as chlorogenic acids or glycosylated flavonoids, are processed enzymatically or by the microbiota into smaller metabolites like dihydroferulic acid (DHFA) and dihydrocaffeic acid (DHCA). Compounds such as epicatechin enter enterocytes via passive diffusion and are intracellularly glucuronidated, with absorption further influenced by basolateral and brush‐border transporters. Approaches like encapsulation have been explored to enhance polyphenol bioavailability.

Complementing this general understanding, a 2024 in vitro study assessed the intestinal absorption of polyphenols from a Spanish tiger nut beverage (TNB) and its by‐product obtained from gastric content processing (TNBP, 10 mL) using Caco‐2 cells, a well‐established model of human intestinal uptake (Llorens et al. 2024). Polyphenols in TNB showed high bioavailability, exceeding 80%, while TNBP‐derived compounds ranged from 61.8% to 83.6%. Trans‐cinnamic acid and epicatechin derivatives were among the most efficiently absorbed, indicating effective transport across the intestinal epithelium. These compounds may help reduce oxidative stress in the gastrointestinal tract and modulate digestive enzyme activity, potentially influencing energy metabolism and nutrient utilization.

A 2024 in vivo study further examined the absorption and bioavailability of polyphenols following acute consumption of pigmented barley and wheat in three healthy adults aged 21–40 (Ed Nignpense et al. 2024). The cereals, grown in Narrabri, Australia, were prepared as a standardized porridge with 200 g of flour, water, milk, sugar, and a pinch of salt. Blood samples were collected at fasting and at 30 min, 1, 2, and 4 h post‐consumption, while urine was sampled at fasting and 4 h to measure phenolic metabolites. Only a small fraction of polyphenols (< 6%) was absorbed within the first 4 h, and plasma caffeic acid was detected inconsistently among participants. Urinary analyses revealed grain‐specific metabolite patterns, with pigmented varieties (purple barley and wheat) showing higher excretion of hippuric, protocatechuic, and caffeic acids. These findings suggest that most cereal‐derived polyphenols reach the colon, where microbial metabolism likely produces bioactive metabolites, potentially contributing to both gut and systemic health.

### 3.1 Polyphenols

Polyphenols are a diverse group of bioactive compounds characterized by multiple hydroxylated aromatic rings, known for their strong antioxidant and anti‐inflammatory properties. Within this class, flavonoids represent a major subclass distinguished by their characteristic structure of two phenyl rings connected by a heterocyclic ring (Kwak and Ju 2017).

These compounds play a crucial role in maintaining intestinal homeostasis by reinforcing the epithelial barrier, modulating immune responses, shaping gut microbiota composition, promoting the growth of beneficial bacteria, and inhibiting pathogenic species (Machado et al. 2021). Beyond their natural activity, in vitro studies demonstrate that polyphenol‐loaded nanocarriers exhibit excellent biocompatibility and efficiently suppress the overproduction of reactive oxygen species (ROS) induced by oxidative stress (Zang et al. 2024).

However, evidence also suggests that under certain conditions, particularly when administered in isolated or soluble forms, polyphenols may exert prooxidant effects, potentially intensifying inflammatory responses in dextran sulfate sodium (DSS)‐induced colitis models associated with colon cancer (Maurer et al. 2020).

#### 3.1.1 Inflammatory Bowel Disease—In Vitro, In Vivo, and Combined Models

Numerous studies demonstrate that plant‐derived polyphenols exert strong antioxidant and anti‐inflammatory effects in models of IBD, both in vitro and in vivo.

Methanolic extract of *Pyrus elaeagnifolia* fruits (100 mg/kg) alleviated acetic acid‐induced colitis in Sprague–Dawley rats by reducing oxidative and inflammatory markers, including malondialdehyde (MDA), nitrite, IL‐6, and TNF‐α, in colonic tissue (Ilhan et al. 2019). Similarly, aqueous extracts of *Aronia mitschurinii* inhibited DSS‐induced colitis in male C57BL/6J mice by suppressing inflammatory pathways (Martin et al. 2018). *Crataegus azarolus* berry extract, rich in polyphenols (107.7 ± 1.43 mg GAE/g), offered dose‐dependent protection (100–400 mg/kg) against acetic acid‐induced UC in Wistar rats through potent antioxidant activity (Sammari et al. 2024).

The ethanolic extract of *Forsythia suspensa* fruits (0.1–0.4 g/mL) improved antioxidant responses and reduced expression of ASC, caspase‐1, IL‐1β, GSDMD, and NLRP3 genes in DSS‐induced UC (Chao et al. 2022). Likewise, *Vaccinium vitis‐idaea* fruit polyphenols inhibited myeloperoxidase activity and decreased cytokine and nitric oxide production (Jeon et al. 2021). *Rubus tereticaulis* ethanol leaf extract (300 mg/kg) also mitigated acetic acid‐induced UC in Wistar rats by enhancing antioxidant and anti‐inflammatory mechanisms (Şen et al. 2023). *Vaccinium corymbosum* aqueous extract (50 mg/kg) improved oxidative balance by lowering COX‐2 and IL‐1β expression in colonic tissues of BALB/c mice (Pervin et al. 2016).

Ethanolic extracts of *Quercus brantii* fruits (200–400 mg/kg) markedly decreased TNF‐α, IL‐6, NO, MPO, and MDA while increasing SOD activity in trinitrobenzene sulfonic acid‐induced UC in rats (Naini, Zargari‐Samadnejad, et al. 2021; Naini, Mehrvarzi, et al. 2021). Comparable protective effects were reported for *Aloe barbadensis Miller* phenolic compounds (Naini, Zargari‐Samadnejad, et al. 2021; Naini, Mehrvarzi, et al. 2021). Similarly, *Brassica oleracea var. capitata rubra* leaf extract (5 mg/kg twice daily), rich in anthocyanins, suppressed pro‐inflammatory cytokine release and reduced colonic inflammation in DSS‐ and TNBS‐induced colitis in mice (Crohn's disease model), though without notable antioxidative activity (Zielińska et al. 2015).

In vitro, *Artemisia argyi* extract (10–40 μg/mL) modulated NF‐κB and Nrf2 signaling in RAW 264.7 cells, while oral administration (200 mg/kg) ameliorated DSS‐induced colitis in mice (Shin et al. 2022). *Aralia elata* aqueous extract demonstrated similar dual effects in vitro (25–75 μg/mL) and in vivo (250–1000 mg/kg), suppressing TNF‐α, IL‐1β, and IL‐6 and improving histological outcomes in DSS‐challenged C57BL/6J mice (Lee et al. 2022).

*Aqueous extracts of Centella asiatica* (tiger grass) reduced IL‐1‐induced inflammation in Caco‐2 cells and enhanced IL‐4 and IL‐10 expression in BALB/c mice, resulting in significant improvement in DSS‐induced colitis (Shin et al. 2025). Similarly, chlorogenic acid from *Lonicera japonica* buds promoted *Lactobacillus* growth and reduced intestinal inflammation, barrier damage, and oxidative stress in DSS‐treated C57BL/6 mice at 200 mg/kg/day (Chen et al. 2024).

Hydroethanolic extract of *Dilodendron bipinnatum* inner stem bark (100–500 mg/kg) reduced MPO activity and GSH depletion, decreased hemorrhage and leukocyte infiltration, and restored mucus production in UC‐induced Wistar rats, without cytotoxic effects in Caco‐2 cells (Oliveira et al. 2021). *Scutellaria baicalensis* ethanol extract (50–200 mg/kg) similarly reduced MPO activity and TGF‐β1 levels in TNBS‐induced colitis in rats, while in vitro (0.05–1 mg/mL) it decreased MDA, CAT, GSH‐Px, and SOD activities in LPS‐stimulated macrophages (Jin et al. 2016).

*Methanolic and aqueous extracts of Cotoneaster integerrimus* twigs and fruits displayed no cytotoxicity (0.1–20 mg/mL) but effectively downregulated pro‐inflammatory biomarkers, reducing H_2_O_2_‐ and LPS‐induced toxicity and TNF‐α expression in HCT‐116 cells (Zengin et al. 2019). The protective compounds, protocatechuic acid, ferulic acid, hesperidin, eriodictyol, and apigenin, were identified as key contributors to its anti‐inflammatory efficacy (Zengin et al. 2019).

Finally, *Mangifera indica* and *Punica granatum* polyphenols act on distinct molecular targets. Mango extracts inhibit the IGF‐1R‐AKT/mTOR pathway, while pomegranate polyphenols downregulate downstream ERK1/2 signaling, jointly reducing inflammation and oxidative stress in UC models (Kim et al. 2016).

Collectively, these findings demonstrate that polyphenol‐rich plant extracts exert protective effects against IBD by modulating oxidative stress, suppressing pro‐inflammatory signaling (NF‐κB, Nrf2, NLRP3, mTOR), enhancing antioxidant defenses (SOD, CAT, GSH), and maintaining intestinal barrier integrity, supporting their therapeutic relevance in ulcerative and CD management.

#### 3.1.2 Colon Cancer—In Vitro, In Vivo, and Combined Models

A broad range of phenolic and polyphenolic plant extracts exhibit significant anticancer potential against CRC through antioxidant, proapoptotic, and cell cycle‐modulating mechanisms.

Phenolic compounds from *Hypericum triquetrifolium* hydroethanolic extract induced apoptosis and cell cycle arrest in HCT‐116 cells at 0.064–0.5 mg/mL (Mahajna et al. 2019). Methanolic seed extracts of *Persea americana* (avocado) demonstrated strong antioxidant and antiproliferative activity against human colon (HT‐29), breast (MCF7), lung (H1299), and prostate (LNCaP) cancer cells at 19–132 μg/mL (Dabas et al. 2019). Similarly, aqueous extracts of *Urtica dioica* leaves inhibited HCT‐116 colorectal carcinoma proliferation and migration at 1–4 mg/mL, highlighting their antibacterial and antioxidant properties (Dakhli et al. 2025).

Ethanolic extracts of *Euphorbia lathyris* seeds significantly reduced the migration of T‐84 and HCT‐15 colon adenocarcinoma cells through caspase activation (3, 8, 9) and autophagy induction (Mesas et al. 2022). Comparable effects were reported for ethanolic extracts of *Moringa oleifera* , *Tropaeolum tuberosum* , and *Annona cherimola* , which suppressed T‐84 and HCT‐15 cell proliferation at 2.5–40 μg/mL via ROS generation and G2/M phase arrest (Fuel et al. 2021).

Phenolic‐rich extracts of *Aronia melanocarpa* leaves inhibited cell migration by up to 86% in HT‐29 and 80% in SW‐480 cells at 50 μg/mL, with stronger effects at 100 μg/mL. The phenolic fraction (360.59 mg/g) reduced MMP‐2 synthesis by up to 72% and 50%, respectively (Owczarek et al. 2024). Likewise, *Glycine max* (soybean) leaf extract inhibited anchorage‐independent growth of HCT‐116 and H1299 cells in a dose‐dependent manner (62.5–500 μg/mL), attributed to its high polyphenol and flavonoid content (Kwak and Ju 2017).

Polyphenolic extracts from *Ribes* spp. (blackcurrant) seeds and young shoots of *Ribes nigrum* , *Vaccinium myrtillus* , and *Vaccinium vitis‐idaea* exhibited potent antioxidant and cytotoxic activity in Caco‐2 and HT‐29 cell lines (Lyashenko et al. 2024; Solcan et al. 2023). *Ribes nigrum* fruit extract induced apoptosis and G0/G1 arrest in HT‐29 and NCM460 cells, reducing MMP‐2 and MMP‐9 expression (Olejnik et al. 2018).

Extracts of *Aristotelia chilensis* fruits achieved complete cytotoxicity against HT‐29 and Caco‐2 cells at > 50 μg/mL while suppressing NO, iNOS, NF‐κB, and COX‐2 expression (Céspedes‐Acuña et al. 2018). *Morus nigra* aqueous and ethanolic extracts showed similar antioxidant and proapoptotic effects, with ROS‐mediated growth inhibition enhanced by heat treatment (Cui et al. 2020). The aqueous extract of *Vaccinium meridionale* induced apoptosis and cell cycle arrest in SW‐480 and SW‐620 cells without mitochondrial disruption (Arango‐Varela et al. 2021).

Curcumin ( *Curcuma longa* ) suppressed adenosine deaminase activity in cancerous and non‐cancerous gastric and colon tissues (Durak et al. 2015a), while *Morchella sextelata* polyphenols inhibited HCT‐116 and HT‐29 proliferation via caspase‐3/9‐ and Bax‐mediated apoptosis, accompanied by ROS accumulation (Zhai et al. 2025). *Punica granatum* (pomegranate) peel methanolic extract reduced colon tumor formation in N‐methyl nitrosourea‐treated rats by downregulating COX‐2, β‐Catenin, and K‐ras, and modulating Wnt/β‐Catenin signaling (Ahmed et al. 2017).

Extracts of *Origanum majorana* showed no acute toxicity up to 10 g/kg but selectively affected cancer cell lines in vitro, with HT‐29 cells being less sensitive than MDA‐MB‐231 (Makrane et al. 2018). *Phlomis fruticosa* and *Phlomis herba‐venti* extracts mitigated oxidative stress, bacterial growth, and colon inflammation in rats, with high phenolic content contributing to LDH reduction and tissue protection (Ferrante et al. 2019).

The ethanolic extract of *Prunus spinosa* inhibited HCT‐116 proliferation in vitro and caused detachment of tumor cells in SCID xenografts (Condello et al. 2019). *Ecklonia cava* aqueous extract reduced CT26 tumor burden in BALB/c mice, enhanced apoptosis, and inhibited metastasis (Gong, Kim, et al. 2023). Similarly, *Thalassia testudinum* hydroethanolic extract suppressed RKO, SW‐480, and CT26 cell growth and activated IFNγ and NFκB signaling in vivo (Hernández‐Balmaseda et al. 2021).

*Artemisia annua* ethanol extract inhibited HCT‐116 proliferation and tumor growth in Balb/c nude mice via PDK1/AKT and mitochondrial apoptosis pathways (Kim et al. 2017), while *Sasa quelpaertensis* ethanol extract targeted colon cancer stem cells through downregulation of CD133, CD44, Notch1, and Sox‐2 (Min et al. 2015).

In murine models, *Euphorbia lathyris* seed extracts (250 mg/kg) enhanced antioxidant enzyme activity (SOD2, GPX2, NFE2L2) (Mesas et al. 2022). Likewise, *Gnetum gnemon* phenolic seed extract induced apoptosis and inhibited colon‐26 tumor growth and metastasis (Narayanan et al. 2015). *Rosmarinus officinalis* polyphenols suppressed colony formation in HT‐29 and SW‐480 cells by ROS‐mediated apoptosis (Pérez‐Sánchez et al. 2019), while *Hippophae rhamnoides* fruit extract regulated miRNA‐mediated apoptosis and cell cycle arrest in colon cancer, both in vitro and in vivo (Wu et al. 2021).

Polyphenolic extracts of *Olea europaea var. sylvestris* induced apoptosis via intrinsic pathways in HCT‐116 and HCT‐8 cells, suppressing tumor growth in xenograft models (Zeriouh et al. 2017). Similarly, *Rhus coriaria* ethanol extract inactivated mTOR and triggered noncanonical autophagy followed by caspase‐7‐dependent apoptosis in HT‐29 and Caco‐2 cells (Athamneh et al. 2017). *Houttuynia cordata* extract decreased PCNA expression and reduced genotoxicity in chemically induced colon tumors (Singai et al. 2024). Extracts from *Vitis* spp. (grape) seeds induced mitochondrial apoptosis in colon cancer stem cells via p53 activation and Bax/Bcl‐2 modulation and reduced β‐catenin‐positive crypts in azoxymethane‐induced colon carcinogenesis (Reddivari et al. 2016).

Overall, these studies demonstrate that phenolic and polyphenolic plant compounds act through multiple molecular mechanisms, including ROS modulation, caspase activation, inhibition of inflammatory mediators, and suppression of metastasis, making them promising natural candidates for CRC prevention and adjunct therapy.

#### 3.1.3 Inflammatory Bowel Disease and Colon Cancer—In Vitro and In Vivo Models

Polyphenol‐rich hydroalcoholic and aqueous extracts of *Allium sativum* (garlic) exhibit potent anti‐inflammatory and anticancer properties relevant to intestinal disorders. In vitro, these extracts suppressed lipopolysaccharide‐induced activation of *TNF‐α*, *NF‐κB*, and *IL‐6* gene expression, thereby inhibiting key pro‐inflammatory signaling pathways (Recinella et al. 2022). Among the tested concentrations (1, 10, and 100 μg/mL), the highest dose significantly decreased the viability of SW‐480 CRC cells.

Ex vivo experiments further confirmed a protective effect on colon tissue from male C57BL/6 mice, suggesting that *A. sativum* polyphenols not only counteract inflammation but may also prevent neoplastic transformation in the colon (Recinella et al. 2022). These findings support the therapeutic potential of garlic‐derived compounds as dual modulators of IBD and colorectal carcinogenesis.

### 3.2 Flavonoids

Flavonoids constitute a diverse class of polyphenolic metabolites widely distributed in plants such as fruits, vegetables, olive oil, and green tea. These compounds are well recognized for their broad biological activities, including antioxidant, anti‐inflammatory, and anticancer properties (Jung et al. 2023; Kölbel et al. 2024).

Among the various subclasses, anthocyanins stand out for their high nutritional and pharmacological value. They exhibit potent antioxidant capacity and have been associated with anti‐inflammatory, antibacterial, neuroprotective, and vision‐preserving functions (Peng et al. 2019). The anthocyanin monomer pelargonidin‐3‐glucoside (P3G) has shown particularly strong redox‐balancing activity, effectively reducing oxidative stress and cellular damage in experimental models (Peng et al. 2019).

Recent investigations highlight that anthocyanin‐rich extracts can modulate inflammation, oxidative imbalance, and microbial dysbiosis in CRC models, under both in vitro and in vivo conditions (Nascimento et al. 2023). These effects are closely linked to their ability to inhibit pro‐inflammatory signaling cascades and restore intestinal barrier function.

Given their combined antioxidant and anti‐inflammatory effects, flavonoids are increasingly considered potential preventive agents in IBD. Epidemiological studies have reported an inverse relationship between flavonoid consumption and IBD severity, with individuals showing milder disease phenotypes consuming higher amounts of these compounds (Kölbel et al. 2024). Furthermore, anthocyanins from natural sources, such as blueberries, purple carrots, black raspberries, and black rice have demonstrated protective activity in DSS‐induced colitis models, where black raspberry anthocyanins notably restored gut microbiota composition (Peng et al. 2019).

Collectively, current evidence underscores the therapeutic relevance of flavonoids, particularly anthocyanins, as multifunctional agents capable of mitigating oxidative stress, suppressing inflammation, and supporting intestinal and systemic health.

#### 3.2.1 Inflammatory Bowel Disease and Colon Cancer—In Vitro, In Vivo, and Combined Models

Numerous plant‐derived extracts display protective effects in IBD through anti‐inflammatory, antioxidant, and mucosal barrier‐stabilizing mechanisms that act on both cellular and molecular levels.

Anthocyanins extracted from *Lycium ruthenicum* (black goji berries) attenuated DSS‐induced colitis in male C57BL/6 mice (200 mg/kg/day) by downregulating pro‐inflammatory cytokines (TNF‐α, IL‐6, IL‐1β, IFN‐γ) and their mRNA expression (Peng et al. 2019). The extract strengthened epithelial tight junctions by upregulating ZO‐1, occludin, and claudin‐1, and simultaneously suppressed NF‐κB activation, restoring intestinal barrier integrity (Peng et al. 2019). Similarly, the hydroalcoholic extract of *Achyrocline satureioides* (1–100 mg/kg) reduced DSS‐induced colonic inflammation in Swiss mice and Wistar rats by elevating IL‐4 and IL‐10 levels while lowering TNF and IL‐6 (Da Silva et al. 2016). Its main flavonoids, luteolin and quercetin, restored mucin production, normalized SOD activity, and decreased lipid hydroperoxide and MPO accumulation (Da Silva et al. 2016).

The anthocyanin‐rich extract of *Prunus mahaleb* fruit activated the Nrf2 pathway, improving antioxidant defenses and alleviating DSS‐induced colitis in BALB/c mice (Ferramosca et al. 2019). Likewise, polyphenolic compounds from *Cyrtocarpa procera* bark extract (200 mg/kg) reduced T‐cell infiltration and cytokine production in female BALB/c mice with DSS colitis, indicating potent anti‐inflammatory and antioxidant activity attributed to flavonoids, such as chrysin, naringenin, kaempferol, and catechin (Rodriguez‐Canales et al. 2020).

*Laurus nobilis* ethanolic leaf extract exhibited dose‐dependent protection in acetic acid‐induced UC. Combined treatment with sulfasalazine (100 and 200 mg/kg) reduced crypt abscesses, inflammatory scores, and colon wet weight in Wistar rats, with flavonoids contributing to elevated SOD and GSH levels and decreased TNF‐α production (Abdullah Al‐Omran et al. 2023). *Filipendula glaberrima* aqueous extract (100–400 μg/mL) inhibited LPS‐induced mediator production in RAW 264.7 macrophages and improved epithelial repair in DSS‐treated C57BL/6N mice, likely through MAPK and NF‐κB pathway inhibition (Jung et al. 2023).

The ethanolic extract of *Camellia sinensis* (green tea) leaves demonstrated antimicrobial and anti‐inflammatory properties. In vitro, it inhibited pathogenic *Escherichia coli* growth, while oral doses of 500–1000 mg/kg suppressed *E. coli* ‐induced colitis by ~90% in BALB/c mice, promoting mucosal healing (Kim et al. 2021). Similarly, flavonoids from the ethanolic extract of *Acanthopanax senticosus* (30–120 mg/L) reduced ROS and MDA levels, while enhancing CAT, SOD, and GPx levels in H_2_O_2_‐challenged macrophages. In vivo, 50–200 μg/L doses prevented DSS‐induced colon shortening and weight loss, underscoring their antioxidant capacity (Su et al. 2022).

The 30% ethanolic extract of *Morus alba* leaves exerted robust anti‐inflammatory and antioxidative effects by reducing cytokine production and ameliorating DSS‐induced colitis symptoms (Lin et al. 2022). Roots of *Ephedra sinica* also showed anti‐inflammatory potential: ethanolic and ethyl acetate extracts (5–320 μg/mL) inhibited NO production in LPS‐stimulated macrophages, while proanthocyanidin fractions (400–800 mg/kg) alleviated DSS colitis by modulating oxidative stress and cytokine activity in male Kunming mice (Lv et al. 2022).

Finally, *Alpinia officinarum* rhizome hexane extract (0.5–10 μg) suppressed HT‐29 cell proliferation and downregulated NF‐κB and COX‐2 mRNA expression (Rajendiran et al. 2018). Rich in galangin, it decreased serum enzyme markers (AST, ALT, ALP, LDH, and γ‐GT) and inflammatory mediators, and at 200 mg/kg reduced colonic inflammation in DSS‐treated Wistar rats (Rajendiran et al. 2018).

Collectively, these studies confirm that polyphenolic, flavonoid, and anthocyanin‐rich plant extracts can protect against colitis and UC by restoring antioxidant balance, inhibiting pro‐inflammatory pathways (NF‐κB, MAPK, TLR‐2/4‐MyD88), and reinforcing intestinal barrier function. Such bioactive preparations hold promise as complementary agents for managing IBD through simultaneous modulation of oxidative and inflammatory mechanisms.

#### 3.2.2 Colon Cancer—In Vitro, In Vivo, and Combined Models

Several plant‐derived extracts have demonstrated cytotoxic, proapoptotic, or antiproliferative properties against CRC cells, often through modulation of oxidative stress and cell death pathways.

The methanolic extract of *Viburnum opulus* fruits exhibited strong prooxidant and cytotoxic effects by elevating intracellular reactive oxygen species (ROS), resulting in DNA fragmentation and apoptosis (Bozali et al. 2020). In LoVo colorectal adenocarcinoma cells, viability declined by 14.9%–52.1% across concentrations of 5–2000 μg/mL, confirming a clear dose‐dependent cytotoxic response (Bozali et al. 2020).

The ethanolic extract of *Origanum majorana* leaves also showed notable anticancer potential, primarily through the induction of autophagy and apoptosis in HT‐29 colon cancer cells (Benhalilou et al. 2019). Exposure to 150 μg/mL reduced cell proliferation, while higher concentrations (300–600 μg/mL) produced a time‐dependent decline in viability and caused G_2_/M phase arrest. These findings suggest that early DNA damage and autophagic activation are key mechanisms in marjoram‐induced cytotoxicity (Benhalilou et al. 2019).

A 2024 investigation on anthocyanin‐rich extracts from *Sambucus nigra* , *Vaccinium myrtillus* , *Rubus fruticosus* , and *Ribes nigrum* revealed that while these preparations were cytotoxic toward cancer cells, they also affected non‐tumorigenic intestinal epithelial cells (Schmutz et al. 2024). In particular, ethanol‐ and DMSO‐based extracts reduced the viability of HCEC‐1CT cells more markedly than anticipated, highlighting potential off‐target toxicity. Among these, *Rubus fruticosus* (blackberry) extract produced strong concentration‐dependent cytotoxicity that synergized with SN‐38, the active metabolite of irinotecan, enhancing its anticancer potency in both HCT‐116 carcinoma and HCEC‐1CT normal colon cells (Schmutz et al. 2024).

Furthermore, methanolic extracts of *Agrimonia pilosa* roots containing quercetin and quercitrin inhibited cell migration and invasion in SW‐480, HCT‐116, and RKO human colon carcinoma lines (Trinh et al. 2022). These effects, mediated via the JNK signaling pathway, were also confirmed in xenograft models, suggesting a potential antimetastatic role for these flavonoid compounds (Trinh et al. 2022).

Overall, these studies indicate that plant extracts from *Viburnum opulus* , *Origanum majorana* , *Agrimonia pilosa*, and anthocyanin‐rich fruits possess potent antitumor activities by promoting oxidative stress, apoptosis, and cell cycle arrest. However, certain preparations may also exhibit cytotoxicity toward healthy intestinal cells, emphasizing the need for targeted formulation and dosage optimization in future CRC therapies.

#### 3.2.3 Inflammatory Bowel Disease and Colon Cancer—In Vitro and In Vivo

The interaction between chronic intestinal inflammation and colorectal carcinogenesis has been widely documented, with several plant‐derived compounds demonstrating the ability to modulate both processes.

The flavone‐rich extract of *Tetrastigma hemsleyani* radix effectively inhibited colorectal tumor development by targeting the Wnt/β‐catenin signaling cascade (Wu et al. 2018). In vitro, treatment of SW‐620 and HT‐29 colon cancer cells led to G0/G1 phase arrest, suppression of epithelial‐mesenchymal transition, and decreased β‐catenin activity, along with the downregulation of its downstream oncogenic proteins (Wu et al. 2018). These effects translated into reduced tumor proliferation and metastatic potential, suggesting that *T. hemsleyani* may serve as a dual‐acting therapeutic agent capable of countering both inflammation‐driven intestinal damage and CRC progression.

### 3.3 Terpenes

#### 3.3.1 Inflammatory Bowel Disease—In Vitro, In Vivo, and Combined Models

Natural extracts rich in bioactive compounds exhibit promising immunomodulatory and barrier‐protective effects in IBD, acting through the regulation of cytokine signaling and oxidative stress responses.

The ethanol extract of *Lindera aggregata* root (0.5–2 g/kg) effectively inhibited the differentiation of CD4^+^ T cells into Th17 cells in vitro and decreased Th17 populations in the mesenteric lymph nodes of treated mice (Lai et al. 2021). Its main constituents, isolinderalactone, linderane, and lindenenol, attenuated intestinal permeability, tissue injury, and disease severity in DSS‐induced UC models. These effects were associated with decreased cytokine production and overall suppression of intestinal inflammation in male C57BL/6 mice (Lai et al. 2021).

Similarly, iridoid glycosides isolated from *Syringa oblata* leaf hydroethanolic extract provided strong protection against DSS‐induced colitis in male Sprague–Dawley rats (Zhang et al. 2020). The extract reduced apoptosis, oxidative stress, and inflammatory cytokine levels by modulating the TLR‐2/4‐MyD88‐NF‐κB pathway. Dose‐dependent responses (20–80 mg/kg) normalized interleukin levels (IL‐2, IL‐4, IL‐5, IL‐13) and improved mucosal recovery (Zhang et al. 2020).

More recently, *Taraxacum officinale* (dandelion) ethanol extract showed pronounced anti‐inflammatory and gut‐protective properties both in vitro and in vivo (Jin et al. 2024). In cell culture (RAW264.7, Caco‐2, NCM460), the extract downregulated pro‐inflammatory mediators, while in male C57BL/6J mice with 3% DSS‐induced UC, oral doses of 25, 50, and 100 mg/kg alleviated clinical symptoms, restored intestinal barrier integrity, balanced gut microbiota composition, and normalized metabolic activity (Jin et al. 2024).

Overall, these findings indicate that *Lindera aggregata*, *Syringa oblata* , and *Taraxacum officinale* extracts exert complementary protective effects against IBD through immunoregulation, suppression of pro‐inflammatory cytokine networks, oxidative stress reduction, and maintenance of epithelial barrier homeostasis.

#### 3.3.2 Colon Cancer—In Vitro, In Vivo, and Combined Models

Natural plant extracts and essential oils display notable cytotoxic and proapoptotic activity against CRC cells, acting through both intrinsic and extrinsic death pathways.

The hexane extract of *Ferula hermonis* root (0–100 μg/mL) inhibited the proliferation of human colorectal (LoVo) and breast adenocarcinoma (MDA‐MB‐231) cells, inducing apoptosis in a dose‐dependent manner (Abutaha et al. 2019). The LoVo line was comparatively less sensitive, with IC₅₀ values of 25 versus 18.2 μg/mL for MDA‐MB‐231, yet still demonstrated marked cytotoxicity and cell death features under microscopic evaluation (Abutaha et al. 2019).

Similarly, *Origanum majorana* essential oil (64–640 μg/mL) significantly restricted HT‐29 human colon cancer cell growth through activation of the p38 MAPK cascade (Athamneh et al. 2020). At 256 μg/mL, colony formation was nearly abolished, and the treatment triggered both mitochondrial (intrinsic) and receptor‐mediated (extrinsic) apoptotic pathways, confirming its dual proapoptotic mechanism (Athamneh et al. 2020).

The oleo‐gum resin essential oil of *Mesua ferrea*, administered at 100 and 200 mg/kg, produced distinct cytotoxic and antimetastatic effects in human colon cancer cell lines (HCT‐116 and LIM1215) in athymic nude mice. Morphological and histological analyses indicated extensive apoptotic and structural alterations within tumor tissues (Asif et al. 2019).

In addition, *Taraxacum officinale* (dandelion) root aqueous extract (0.5–6 mg/mL) promoted cell death in over 95% of colon carcinoma cells, HT‐29 (p53‐mutant) and HCT‐116 (p53 wild‐type), within 48 h, by activating multiple apoptotic signaling cascades (Ovadje et al. 2016). In vivo, oral administration (40 mg/kg/day) was non‐toxic in Balb/c and CD‐1 nu/nu mice and effectively suppressed colon tumor growth in xenograft models (Ovadje et al. 2016).

Collectively, these studies confirm that phytochemical extracts from *Ferula hermonis*, *Origanum majorana* , *Mesua ferrea*, and *Taraxacum officinale* can induce apoptosis, inhibit tumor proliferation, and reduce metastatic potential in CRC models, supporting their promise as natural anticancer agents.

### 3.4 Multiple Classes of Compounds

#### 3.4.1 Inflammatory Bowel Disease—In Vitro, In Vivo, and Combined Models

A wide range of botanical and marine extracts have demonstrated therapeutic potential against IBD through antioxidant, anti‐inflammatory, and immunomodulatory mechanisms in both in vitro and in vivo models.

Aqueous extracts from *Taraxacum campylodes* , *Coix lachryma‐jobi*, *Smilax glabra*, *Sanguisorba officinalis* , *Styphnolobium japonicum* , *Prunus persica* , *Sophora flavescens* , and *Eupolyphaga sinensis*, rich in matrine and oxymatrine, suppressed HT‐29 cell proliferation (~50% viability at 5 mg/mL) and attenuated DSS‐induced colitis in C57BL/6J mice at 1–4 g/kg by reducing oxidative and inflammatory responses (Fang et al. 2018). A synergistic protective effect was also reported when *Cornus mas* ethanolic extract (20–100 mg/kg) was combined with sulfasalazine, enhancing Th17/Treg regulation in Wistar rats (Szandruk‐Bender et al. 2023). Similarly, a mixture of bamboo shoot and *Artemisia capillaris* extracts alleviated DSS‐induced colitis in C57BL/6N mice, improving colon shortening, histological integrity, and disease activity scores, while reducing ROS in HT‐29 and Caco‐2 cells (Kim et al. 2022).

Several individual extracts have shown potent anti‐inflammatory and antioxidant actions. *Artemisia ordosica* aqueous extract (0.5–10 mg/mL) enhanced enzymatic antioxidant defenses, decreased TNF‐α, IL‐6, and MDA levels, and modulated gut microbiota and bile acid metabolism to restore intestinal homeostasis in mice (Jiang et al. 2025). *Origanum majorana* flower ethanol extract, alone or with sulfasalazine (500 and 100 mg/kg/day), downregulated NF‐κB activity and cytokine transcription, reducing UC severity in rats (Taha et al. 2023). *Manilkara zapota* leaf extract (50–200 mg/kg) ameliorated DSS‐induced inflammation in BALB/c mice by suppressing TNF‐α and IL‐6, enhancing SOD levels, and improving mucosal architecture (Tamsir et al. 2024).

Other studies confirmed similar effects in different plant models. *Lens culinaris* var. Eston green extracts (0.25–10 μg/mL) enhanced cell viability, inhibited TLR‐4 and iNOS expression, lowered IL‐1, and increased IL‐10, promoting an anti‐inflammatory balance (Panaro et al. 2024). *Alstonia boonei* stem bark extracts (125–250 mg/kg) reduced TNF‐α, IL‐6, IL‐1β, and PGE₂ levels, increased SOD, CAT, and GSH, and decreased MDA and nitric oxide in DSS‐induced colitis (Adjouzem et al. 2020). *Alnus japonica* bark ethanol extract (30–60 mg/kg in vivo; 30–60 μg/mL in vitro) attenuated colitis symptoms and prevented epithelial barrier loss by inhibiting IL‐1β, IL‐6, TNF‐α, COX‐2, and maintained ZO‐1 and occludin expression (Chi et al. 2018).

Phenolic‐rich plants such as *Thymus longicaulis* subsp. *chaubardii* and *Rosmarinus officinalis* also showed strong antioxidant and anti‐inflammatory activity. The former demonstrated ABTS and DPPH radical scavenging, LOX‐5 inhibition, and cytokine modulation (Ozbeyli et al. 2025), while the latter (120 mg/mL) reduced UC lesions more effectively than sulfasalazine in Wistar rats (Yilmaz et al. 2022). *Rosa roxburghii* ethanolic extract (200–500 μg/mL in vitro; 300–500 mg/kg in vivo) protected the intestinal barrier via IL‐17/TNF pathway modulation and gut microbiota regulation, increasing beneficial genera such as *Ruminococcus* and *Parabacteroides* (Li et al. 2023). *Thuja occidentalis* extract (25–50 mg/kg) further alleviated TNBS‐induced colitis by decreasing IL‐6 and TNF‐α, restoring mucosal structure, and reducing lipid peroxidation (Stan et al. 2019).

Marine and herbal sources have also produced encouraging outcomes. Polysaccharides, terpenes, and flavonoids from *Caulerpa mexicana* (2 mg/kg/day) alleviated UC symptoms in BALB/c mice, decreasing Th1‐ (IFN‐γ, IL‐12, TNF‐α) and Th17‐type (IL‐6, IL‐17) cytokines (Bitencourt et al. 2015). *Bletilla striata* aqueous extract (1–4 g/kg) ameliorated DSS‐induced colitis in C57BL/6 mice by modulating the EGFR/PI3K/AKT pathway and suppressing IL‐6 and TNF‐α (Gong, Lv, et al. 2023).

Additional plant species demonstrated complementary or mechanistic effects. *Morinda officinalis* extract (20–80 mg/kg/day) alleviated DSS‐induced chronic colitis in Kunming mice by regulating inflammatory cytokines (TNF‐α, IL‐6, IL‐17) and lymphocyte apoptosis (Liang et al. 2017). *Laurus nobilis* leaf extract (200 μL) reduced epithelial injury, colon shortening, and CD4^+^ infiltration by inhibiting NF‐κB activation (Correa and Orlando 2023). *Gleditsia sinensis* methanol extract mitigated DSS‐induced inflammation by reducing IL‐1β, TNF‐α, and IFN‐γ; combined with *Lactobacillus brevis* KY21, it further inhibited p65 nuclear translocation and supported mucosal recovery (Kim et al. 2015). Finally, *Ilex rotunda* aqueous extract (1.8 g/kg in vivo; 120 μg/mL in vitro) restored mucosal barrier function and downregulated oncostatin M signaling in colitis models (Li et al. 2024).

Collectively, these studies reveal that a wide array of plant and marine bioactive compounds attenuate intestinal inflammation by reducing oxidative stress, modulating cytokine signaling (NF‐κB, EGFR/PI3K/AKT, Th17/Treg), preserving epithelial barrier integrity, and rebalancing gut microbiota. Their consistent effects across models support their potential as adjunct therapies for IBD management.

#### 3.4.2 Colon Cancer—In Vitro, In Vivo, and Combined Models

A growing body of research highlights the ability of plant and fungal extracts to counteract colorectal carcinogenesis by influencing oxidative stress, inflammation, and programmed cell death.

In vivo findings show that *Crataegus pinnatifida* aqueous extract alleviated DSS‐induced intestinal damage in Balb/c mice through suppression of IL‐6, IL‐8, IL‐17, and IL‐1β, stimulation of caspase‐3 activity, and normalization of the gut microbiota (Ma et al. 2023). Likewise, oral administration of *Moquiniastrum polymorphum* ssp. *floccosum* extract (100 mg/kg) exhibited preventive and protective activity against 1,2‐dimethylhydrazine‐induced CRC without evident systemic toxicity (Limeiras et al. 2017). Treatment with *Strobilanthes crispus* extract (250–500 mg/kg) diminished azoxymethane‐related precancerous lesions and oxidative damage while enhancing the antioxidant defense system in rats (Al‐Henhena et al. 2015). Similarly, *Annona reticulata* leaf preparations reduced tumor burden and histological dysplasia in experimental colon cancer models by restoring SOD, CAT, and GSH enzyme levels (Khan et al. 2021).

In vitro investigations further confirm the antiproliferative capacity of many natural extracts. *Prunella vulgaris* ethanol extract (0.25–1.5 mg/mL) limited HCT‐8 colon carcinoma cell expansion through miR‐34a activation and suppression of *Notch1*, *Notch2*, and *Bcl‐2* (Fang et al. 2017). *Sargassum ilicifolium* ethanolic and chloroform fractions significantly decreased HT‐29 cell viability (IC₅₀ = 1415 μg/mL) (Sumithra et al. 2017). In contrast, *Tribulus terrestris* fruit methanol extract (0.5–5 μg/mL) showed non‐selective toxicity toward prostate, colon, and fibroblast‐like cells (Pourali et al. 2016).

Extracts of *Inonotus obliquus* (2.5–10 μg/mL) interfered with the cell cycle machinery of HT‐29 cells by lowering *CDK2*, *CDK4*, and *cyclin D1* expression and upregulating *p21*, *p27*, and *p53* (Lee et al. 2015). *Annona muricata* ethyl acetate fruit extract suppressed HT‐29 cell proliferation and altered cycle progression dynamics (Daddiouaissa et al. 2021), while *Salvia officinalis* essential oil inhibited the growth of Caco‐2 and HCT‐116 cells by extending the G0/G1 phase (Luca et al. 2020).

Polyphenolic‐rich extracts from *Cornus mas* and *Celtis aetnensis* reduced viability and triggered apoptosis or necrosis in colon cancer cells, depending on concentration (Acquaviva et al. 2016; Blagojević et al. 2021). *Acmella caulirhiza* leaf and flower preparations (5–500 μg/mL) promoted programmed cell death through caspase‐3 activation in MC‐38 cells (Tienoue Fotso et al. 2024). Extracts of *Malus* spp. (30 mg/mL) also decreased HT‐29 and U‐87 cell survival while displaying antioxidant and hyaluronidase‐inhibitory properties (Butkeviciute et al. 2021).

Among fungi, *Ganoderma lucidum* spore extracts (1.6–10 mg/mL in vitro; 75–150 mg/kg in vivo) regulated genes associated with growth control and apoptosis, including *p21*, *p16*, *Bcl‐2*, and *Bax* (Li et al. 2017). *Inula viscosa* aqueous leaf extract (150–300 mg/kg) restricted MC‐38 tumor progression in mice (Bar‐Shalom et al. 2019). *Heterobasidion annosum* methanolic extract (0.1–5 μg/mL) markedly reduced DLD‐1 cell proliferation and tumor volume through NF‐κB and p53 activation and acted synergistically with 5‐fluorouracil (Sadowska et al. 2023, 2020).

Herbal combinations such as *Astragalus mongholicus* and *Curcuma aromatica* (0.32–1.28 g/kg/day) restored microbial homeostasis, enhanced short‐chain fatty acid synthesis, and limited colon tumor advancement via *Cyclin D1* and *C‐myc* suppression (Gu et al. 2021). *Hedyotis diffusa* ethanol extract impaired colon cancer cell proliferation and interfered with multiple signaling cascades, including AKT, MAPK, and STAT3 (Feng et al. 2017). *Gnetum montanum* (10–120 μg/mL; 28–56 mg/kg/day) selectively constrained SW‐480 tumor growth and metastatic potential without cytotoxicity toward normal cells (Pan et al. 2022). *Portulaca oleracea* aqueous and hydroalcoholic extracts (0.07–2.25 μg/mL) disrupted the Notch/β‐catenin axis in colon cancer stem cells (Jin et al. 2017).

Other phytocompounds have displayed complementary activity. *Curcuma longa* ethanolic extract (200 mg/kg) reduced colon tumor recurrence in patient‐derived xenografts, likely through PI3K‐AKT pathway modulation (Li et al. 2021). *Caulis spatholobi* ethyl acetate extract (25–400 μg/mL in vitro; 200 μL in vivo) interfered with metastatic processes by reducing tumor‐associated platelet aggregation (Sun et al. 2020). *Tripterygium wilfordii* methanol extract (0–500 μg/mL) limited HTB‐39 colon cancer cell proliferation and altered cell cycle regulation (Zhu et al. 2018).

Additionally, the lipid‐enriched extract of *Persea americana* seed (20–150 μg/mL) activated caspase‐8 and ‐9 pathways, elevated mitochondrial ROS, and modulated cytokine secretion profiles (Lara‐Márquez et al. 2020). *Rheum rhabarbarum* aqueous extract, rich in anthraquinones, decreased adenosine deaminase activity in both malignant and normal intestinal tissues (Durak et al. 2015b). Finally, *Scaphium affine* ethanol extract containing β‐sitosterol (75–125 mg/mL in vitro; 75–100 mg/kg/day in vivo) triggered detachment‐related cell death by inhibiting EGFR/AKT signaling in HCT‐116 xenografts (Kawk et al. 2021).

## 4 Conclusions

In the process of synthesizing the literature for this review, it became increasingly evident that plant‐derived bioactive compounds hold substantial therapeutic potential in the context of intestinal health. Polyphenols, flavonoids, and terpenes found in many medicinal plants demonstrate the ability to modulate oxidative stress and inflammation, two major drivers of both IBD and colorectal carcinogenesis. These natural compounds not only suppress pro‐inflammatory cytokines and regulate oxidative pathways but also contribute to maintaining the structural and functional integrity of the intestinal barrier.

Although the majority of available evidence is derived from in vitro and in vivo studies, the overall coherence of findings across species and experimental approaches strengthens the biological plausibility of these phytochemicals. Nevertheless, it also became clear throughout this review that the translation of these results to clinical applications remains a major challenge. Determining the effective doses, understanding long‐term safety, and assessing interactions with conventional therapies are essential steps for future research.

This review brings together recent scientific advances to provide an integrated overview of plant species and phytochemicals with demonstrated antioxidant, anti‐inflammatory, and anticancer properties. By consolidating this evidence, it aims to guide researchers toward identifying promising candidates for further pharmacological evaluation and clinical validation. Ultimately, the growing intersection between traditional medicine and modern food science opens new directions for developing functional foods and nutraceuticals designed to prevent or mitigate chronic intestinal diseases.

## Author Contributions

O.‐R.H.: conceptualization. O.‐R.H.: data curation. O.‐R.H.: methodology. O.‐R.H.: investigation. O.‐R.H.: writing – original draft preparation. O.‐R.H.: writing – review and editing. O.‐R.H. and S.A.: visualization. O.‐R.H.: supervision. S.A.: all authors have read and agreed to the published version of the manuscript.

## Funding

The authors have nothing to report.

## Ethics Statement

The authors have nothing to report.

## Consent

The authors have nothing to report.

## Conflicts of Interest

The authors declare no conflicts of interest.

## Data Availability

The data that support the findings of this study are available from the corresponding author upon reasonable request.
